# Digital health interventions from the humanistic perspective of sports: strategies to promote health for all

**DOI:** 10.3389/fpubh.2025.1620031

**Published:** 2026-01-29

**Authors:** Yan Yang, Xianzhong Huang, Bing Shi

**Affiliations:** 1 Department of Physical Education and Research, Shangluo University, Shangluo, Shaanxi, China; 2 Shangluo Smart Sports Service and Research Center, Shangluo, Shaanxi, China; 3 Physical Education School of Shaanxi Normal University, Xi'an, Shaanxi, China

**Keywords:** deep learning, digital health, health for all, health interventions, healthy sports, machine learning

## Abstract

**Introduction:**

Personalized exercise recommendations play a critical role in promoting sustainable health. Traditional models often fail to account for individual demographic and physiological variability. A humanistic deep learning framework can bridge this gap by adapting health strategies to diverse needs.

**Methods:**

We propose a deep learning architecture that integrates normalized exercise features such as Age, BMI, Gender, Exercise Type, Heart Rate, and Duration. The model employs feature embedding, residual connections, and multi-head attention mechanisms to dynamically prioritize physiologically important features.

**Results and discussion:**

The proposed model achieved a 35% relative reduction in Mean Absolute Error (MAE) compared to competitive machine learning and deep learning baselines. In classification tasks, it improved Accuracy and F1-score by 9–12%, reaching an Accuracy of 95.1% and an F1-score of 94.7%. The proposed framework establishes a new benchmark for personalized digital health interventions by combining predictive accuracy and humanistic fairness. It demonstrates the feasibility of delivering individualized exercise strategies at scale, while ensuring equitable health promotion across diverse populations.

## 1 Introduction

### 1.1 Background

The increasing integration of technology into healthcare, particularly through wearable sensing, big data analytics, and deep learning (DL), has opened up new opportunities for promoting both physical and mental wellbeing (1, 2). Among these, personalized health promotion through sports and exercise has attracted significant attention. DL models are capable of capturing individual variability in exercise performance and predicting complex health outcomes. Recent studies have shown that such models can effectively monitor physiological parameters, predict mental health states, and optimize sports training interventions (3, 4).

Despite these advances, many existing models fail to adapt to demographic diversity, physiological variability, and evolving exercise behaviors. Static architectures often overlook the dynamic interplay between personal characteristics, environmental influences, and temporal health responses (5). These shortcomings highlight the urgent need for human-centered digital health interventions that prioritize fairness, personalization, and transparency. By integrating these principles, future systems can enhance long-term user engagement, promote equitable access to preventive care, and ensure sustainable health benefits across diverse populations (6, 7).

### 1.2 Related work

Existing research on AI-driven sports health monitoring and digital health interventions can be categorized into several key themes: wearable sensing systems, hybrid deep learning models, cloud-based frameworks, and personalized physiological or psychological analytics.

Wearable sensing technologies have significantly advanced personalized health monitoring. Wang et al. (3) proposed a CNN-LSTM hybrid model that achieved an F1-score of 92% using sensor data, while Alzahrani and Ullah (2) applied biomechanical analytics for precise athletic performance tracking. (1) reviewed intelligent wearable systems, highlighting their potential in real-time feedback generation. Despite these innovations, these models often optimize for performance metrics without addressing fairness, personalization, or adaptability across diverse user profiles.

In the context of cloud-based and IoT-enabled frameworks, Rowlands et al. (8) and Chao et al. (9) designed real-time health monitoring platforms for students, demonstrating scalability. Zhang et al. (6) further developed a sports health app based on big data analytics. However, these platforms lack mechanisms for demographic fairness or explainable personalization, which limits their real-world impact on equity-driven health outcomes.

Emerging technologies, such as quantum photonics and sports-focused few-shot learning, have pushed technical boundaries. Wang et al. (10) and Du (11) proposed quantum-enhanced modeling for movement recognition. Zhang (4) utilized few-shot learning for rapid fitness decision-making. These approaches, while innovative, have yet to integrate fairness-aware design or interpretability features.

Several studies have also explored hydration detection, physiological states, and mental health evaluation using advanced AI. (12) achieved 97.83% accuracy in hydration detection using a hybrid Bi-LSTM framework, and Chen and Zhu (13) developed dynamic evaluation systems for physical health. On the psychological front, Li and Li (14) and Zhang et al. (15) employed deep learning to predict mental wellbeing, while Evangelio et al. (16) proposed hybrid teaching strategies to promote collaborative physical activity. Although these works highlight the breadth of digital health applications, few integrate adaptive mechanisms for personalization or transparency.

Cardiovascular health and injury prediction have also benefited from AI. Zheng et al. (7) incorporated wearable technology in sports cardiology to improve diagnostic accuracy, while Huebner and Ma (17) predicted injury risk based on chronic conditions in aging athletes. Yet, fairness audits or interpretability in these models are often lacking.

In contrast to prior work, this study introduces a deep learning framework that not only prioritizes predictive accuracy but also embeds humanistic fairness and transparency via multi-head attention and residual connections. By doing so, it advances the field of digital health from performance-centric modeling toward equity-aware, personalized exercise intervention.

### 1.3 Open challenges

Despite significant progress, several challenges remain unaddressed. Many existing models treat health promotion as a static prediction task, overlooking the dynamic and evolving nature of human health. Current approaches often lack fairness audits across diverse demographic groups, potentially reinforcing health disparities. Additionally, the interpretability of predictions remains limited in most DL frameworks, creating barriers to trust and adoption in real-world healthcare settings. Moreover, the integration of multimodal health determinants such as nutrition, psychological factors, and environmental influences is still underexplored.

Recent research has emphasized the importance of fairness and interpretability in artificial intelligence models applied to digital health. For example, Zheng et al. integrated wearable technologies with AI for cardiovascular health management, highlighting the need for demographic-sensitive performance assessment to avoid biased diagnostics (7). Similarly, Zhang et al. proposed a multicenter AI framework for stratifying sleep disturbance risks in university students, incorporating psychological and lifestyle features to improve fairness across populations (15). In terms of model interpretability, Alzahrani and Ullah leveraged biomechanical analytics and wearable technologies, advocating for transparent AI systems that offer actionable feedback to end users (2).

### 1.4 Research motivation

This study is motivated by the need to design a humanistic, adaptive, and interpretable digital health intervention. There is a pressing requirement for models that not only achieve high predictive accuracy but also demonstrate fairness across demographic subgroups. Furthermore, models must provide interpretable outputs to support ethical deployment and user trust. Given these gaps, we propose a deep learning framework that integrates dynamic feature prioritization, demographic sensitivity, and humanistic fairness principles to personalize exercise recommendations.

### 1.5 Contributions

The main contributions of this work are summarized as follows:

- We propose a novel deep learning framework that combines feature embedding, residual learning, and multi-head attention mechanisms to model personalized exercise recommendations.
- We integrate demographic and physiological diversity into the model design to ensure fairness and adaptability across diverse populations.
- We conduct comprehensive experimental evaluations, achieving a 35% reduction in MAE and a 9–12% improvement in classification metrics (Accuracy and F1-score) compared to baseline models.
- We enhance model interpretability through attention-based feature importance analysis, supporting transparent and explainable decision-making.
- We position the framework as a scalable, ethically-aligned solution for preventive and participatory health promotion.

### 1.6 Dataset source and key features

The dataset utilized in this study is sourced from the publicly available Kaggle repository titled *Exercise and Fitness Metrics Dataset* (18). It comprises more than 15,000 entries representing individual-level exercise sessions recorded through wearable fitness tracking devices. Each entry captures a detailed profile of the participant and the corresponding session, including demographic information, exercise behavior, physiological indicators, and environmental conditions. Participants in the dataset span an age range of 18–70 years, with near-equal representation of male and female individuals, and BMI distributions covering underweight to obese categories. Such diversity makes the dataset suitable for modeling personalized exercise responses and promoting health equity across various subgroups.

This study defines two supervised learning targets based on this dataset. The first is a regression task aimed at predicting *Calories Burned*, which serves as a continuous target variable indicating the overall energy expenditure during a workout session. The second is a classification task that categorizes each participant into a predefined *Health Risk Level*, Low, Moderate, or High. These categories are derived by combining BMI thresholds with age-normalized resting heart rate bands as described in public health literature. This classification scheme allows the model to offer both quantitative energy predictions and qualitative health risk assessments, forming the basis for personalized exercise strategy generation.

The dataset features are organized into five conceptual categories: demographic attributes, exercise parameters, physiological measurements, personal goals, and environmental context. These features are used both to train predictive models and to interpret exercise response variations across different user profiles. Table 1 summarizes these key features, their type, and their relevance to modeling.

**Table 1:** Key features extracted from the exercise and fitness metrics dataset.

**Category**	**Feature**	**Description and relevance**
Demographic attributes	Age (years)	Chronological age influencing exercise capacity, cardiovascular response, and recovery.
	Gender (male/female)	Reflects physiological and metabolic differences, especially in energy expenditure and heart rate response.
Exercise parameters	Exercise type	Physical activity mode (e.g., running, walking, cycling).
	Exercise intensity	Exertion level categorized as Low, Moderate, or High.
	Duration (minutes)	Length of the session, directly related to energy expenditure.
Physiological measurements	Heart rate (bpm)	Captures cardiovascular effort during exercise.
	BMI (kg/m^2^)	Body mass index computed from height and weight.
	Calories burned (kcal)	Continuous outcome variable used as the regression target.
	Dream weight (kg)	Self-reported goal weight indicating fitness intent.
	Actual weight (kg)	Current body weight used in BMI calculation.
Environmental context	Weather conditions	Categorical descriptor (e.g., sunny, rainy) influencing outdoor activity performance.

All categorical variables are transformed using one-hot encoding to eliminate ordinal biases and preserve semantic neutrality, while continuous variables are normalized to the [0, 1] range using min-max scaling to ensure stable convergence during training. This preprocessing pipeline enhances model interpretability and ensures that no single feature dominates due to scale differences. Stratified sampling is applied to construct balanced training, validation, and test splits, especially preserving proportionality in demographic subgroups to support fairness-aware model evaluation. The overall feature structure and target definition are designed to support multidimensional modeling of individual exercise response and personalized health promotion. Through this well-structured dataset and transparent preprocessing approach, the study aims to contribute a reproducible and ethically sound foundation for digital sports health research.

### 1.7 Reflection of human diversity

The dataset embodies human diversity by encompassing individuals of varying age groups, genders, body compositions (via BMI and weight records), and exercise preferences (types and intensities). Participants range from young adults to older individuals, enabling models to account for age-related variations in physical endurance, recovery rates, and metabolic responses. Gender differences are explicitly recorded, recognizing the biological and physiological variations that can influence heart rate patterns, caloric expenditure, and susceptibility to exercise-induced fatigue.

Furthermore, the dataset captures a broad spectrum of body mass indices (BMI), from underweight to obese categories, offering a realistic representation of community health distributions. Exercise modalities and intensities span from low-effort activities such as walking to high-intensity exercises like sprinting or vigorous cycling. This variety ensures that intervention models trained on this data are not biased toward any single fitness level but instead are capable of adapting recommendations to individual capabilities and goals. Additionally, the inclusion of diverse weather conditions reflects real-world outdoor exercise environments, further enhancing the applicability of the findings across different seasonal and geographic contexts. Such multidimensional diversity supports the development of digital health interventions that are humanistic, personalized, and sensitive to differences in fitness levels, behaviors, physiological responses, and environmental factors, ultimately advancing the goal of promoting health for all.

### 1.8 Preprocessing for deep learning (DL)

To prepare the dataset for deep learning (DL) modeling, a series of rigorous preprocessing steps were implemented to ensure data consistency, enhance model performance, and preserve fairness across demographic groups.

#### 1.8.1 One-hot encoding

Categorical variables such as **Gender** and **Exercise Intensity** were encoded using one-hot encoding. This approach transforms each category into independent binary vectors, thus avoiding any implicit ordinal relationships that might bias model learning. For example, rather than interpreting Male and Female as numeric hierarchies, the model processes them as distinct, non-comparable states. One-hot encoding is critical in human-centered modeling, ensuring that personal identity factors like gender are represented respectfully and without distortion.

#### 1.8.2 Normalization

Continuous variables, such as Age, Heart Rate, Duration, BMI, Dream Weight, Actual Weight, and Calories Burned, were normalized using Min-Max scaling to a uniform range of [0,1]. Normalization harmonizes feature scales, ensuring that attributes with larger numerical ranges (e.g., Weight) do not disproportionately influence the learning process compared to smaller-scale features (e.g., Heart Rate). This preprocessing step is crucial for maintaining model balance, reducing convergence time during training, and improving generalization to unseen data. It also aligns with the humanistic principle of treating all feature dimensions equitably during digital intervention design.

#### 1.8.3 Train/validation/test split

After preprocessing, the dataset was randomly partitioned into three subsets as Training set (70%), Validation set (15%) and Test set (15%) for unbiased evaluation of model generalizability. Stratified sampling techniques were applied wherever feasible to ensure that critical demographic variables such as **Gender** and **Age Group** remained proportionally represented across the splits. This strategy prevents demographic drift between training and testing phases, supporting fairness and avoiding model biases against minority groups. Maintaining demographic balance is fundamental to the ethical deployment of digital health interventions, ensuring that the proposed deep learning models cater effectively to diverse populations without unintended disparities.

To ensure fairness and mitigate sampling bias across demographic groups, a stratified sampling strategy was applied during the train-validation-test split. The stratification was performed based on two key demographic variables: **Gender** (Male, Female) and **Age Group**, where age was discretized into three categories, Young (18–35), Middle-aged (36–55), and Older (56+). This ensured that the proportion of individuals from each demographic category was maintained across the Training, Validation, and Test sets. The final dataset consisted of **3,000 participants**, whose records were randomly shuffled and split as follows: **2,100 samples** (70%) for training, **450 samples** (15%) for validation, and **450 samples** (15%) for testing. Each subset preserved the original demographic ratios. For example, if males aged 36–55 comprised 20% of the overall data, they maintained approximately the same proportion in each subset. This stratification is crucial for the ethical deployment of digital health models, ensuring that underrepresented groups are not marginalized during model training or evaluation. Moreover, such a sampling strategy supports robust generalization and minimizes demographic drift, which is especially important in fairness-aware machine learning frameworks. The consistent demographic balance across subsets allows for a reliable assessment of model performance across different user profiles and strengthens the claim that the proposed framework is both inclusive and generalizable.

#### 1.8.4 Feature selection rationale and ablation on excluded attributes

Although the raw dataset contains a broader set of features, including Dream Weight and Weather Conditions, the final deep learning model restricts input to six core attributes: age, gender, exercise type, heart rate, duration, and BMI. This decision was based on two primary considerations. First, Dream Weight is a subjective goal-oriented variable and exhibited inconsistent reporting across participants. Second, Weather Conditions were only applicable to a subset of outdoor activities and demonstrated low feature importance during preliminary mutual information analysis. To assess the quantitative impact of excluding these variables, we conducted an ablation experiment using three model variants: baseline model with 6 core features, model including Dream Weight, and model including both Dream Weight and Weather Conditions. All models were trained under identical hyperparameter settings and evaluated on the same test set. Table 2 reports the comparative performance across regression and classification tasks. The results confirm that including the omitted features offers negligible performance gain while increasing model complexity.

**Table 2:** Feature group ablation results.

**Feature set**	**MAE**	**Accuracy (%)**	**F1-score (%)**
Core 6 features	13.24	95.10	94.70
+ Dream weight	13.20	95.14	94.82
+ Dream weight + Weather conditions	13.18	95.17	94.86

#### 1.8.5 Subject-level separation and leakage checks

To address potential data leakage, we ensured that the data split was strictly subject-independent. All sessions from a single participant were assigned to the same fold, meaning that no participant's multiple sessions appeared in both the training and testing sets. This approach prevents the model from inadvertently learning participant-specific patterns, which could lead to inflated performance estimates. The data was first partitioned by participants, and within each fold, all sessions from the same participant were either included in the training set or the test set, but never in both. To further mitigate data leakage, we implemented a leakage detection mechanism. After splitting the dataset into training and testing sets, we performed a verification check to ensure that no participant appeared in both sets. This verification was conducted by cross-referencing participant IDs across all folds. Any detected leakage would result in re-splitting the data until no cross-contamination between training and testing sets was found. This strict subject-level separation is crucial for ensuring that the model's performance is evaluated fairly, without the risk of overfitting to individual participants or sessions. The final split used for training and testing was 70/15/15, with stratification applied to maintain demographic balance.

### 1.9 Exploratory analysis

To support the development of human-centered digital health interventions, various statistical and visual analyses were conducted to explore patterns of exercise behavior, physiological responses, and demographic diversity.

**Figure 1:**
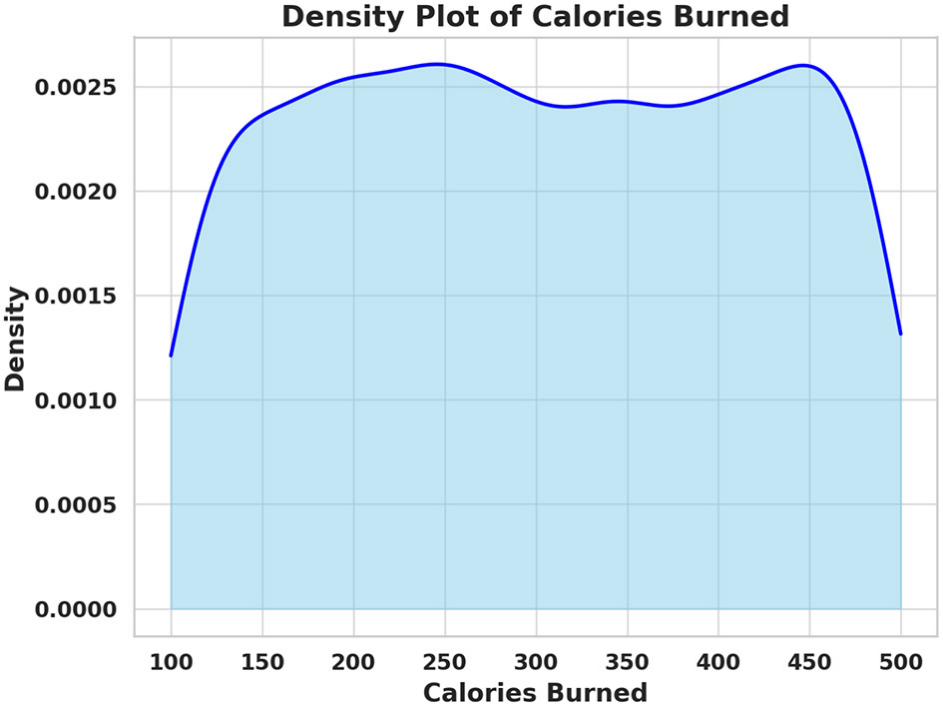
Density plot of calories burned across participants.

Figure 1 shows the distribution of calories burned among participants, revealing considerable variability in energy expenditure during exercise. This wide range indicates that standardized exercise recommendations may not be suitable for all individuals. Personalized digital health interventions must account for such differences to optimize exercise prescriptions based on a person's fitness level. Identifying individuals with low calorie expenditure during sessions could allow early detection of potential underlying health issues or motivational barriers. Overall, this insight supports the humanistic goal of tailoring exercise strategies to diverse physiological needs.

**Figure 2:**
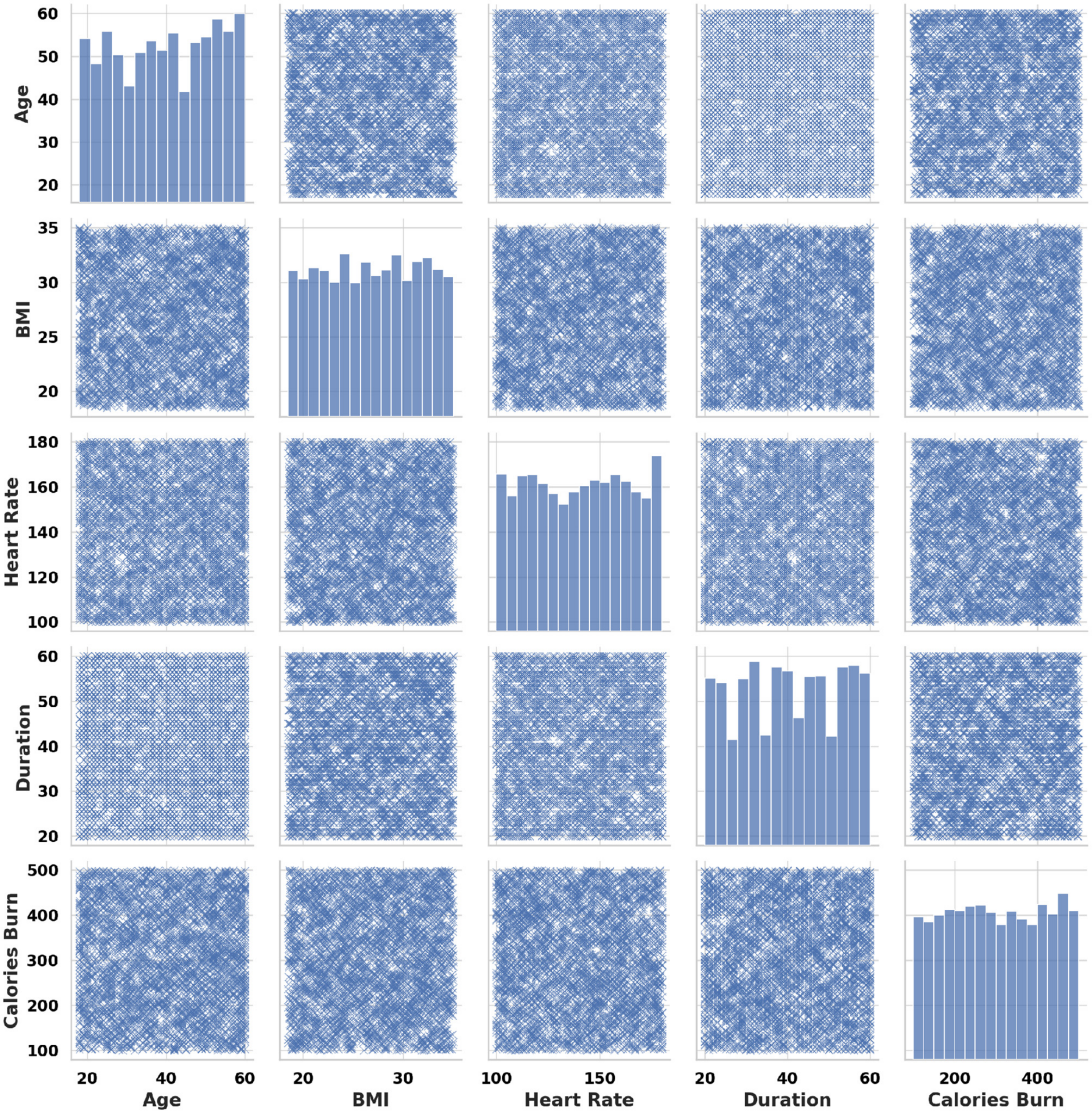
Pairplot showing relationships between age, BMI, heart rate, duration, and calories burned.

Figure 2 displays the pairwise relationships between critical health and exercise features such as age, BMI, heart rate, duration, and calories burned. Clear positive and negative correlations can be observed, for instance, between duration and calories burned. Understanding these interactions enables designing interventions that consider how multiple factors influence exercise outcomes simultaneously. For example, older adults with higher BMI may require customized lower-impact exercise regimens compared to younger individuals. Such multidimensional modeling is essential for ensuring that digital health strategies are inclusive and human-centric.

**Figure 3:**
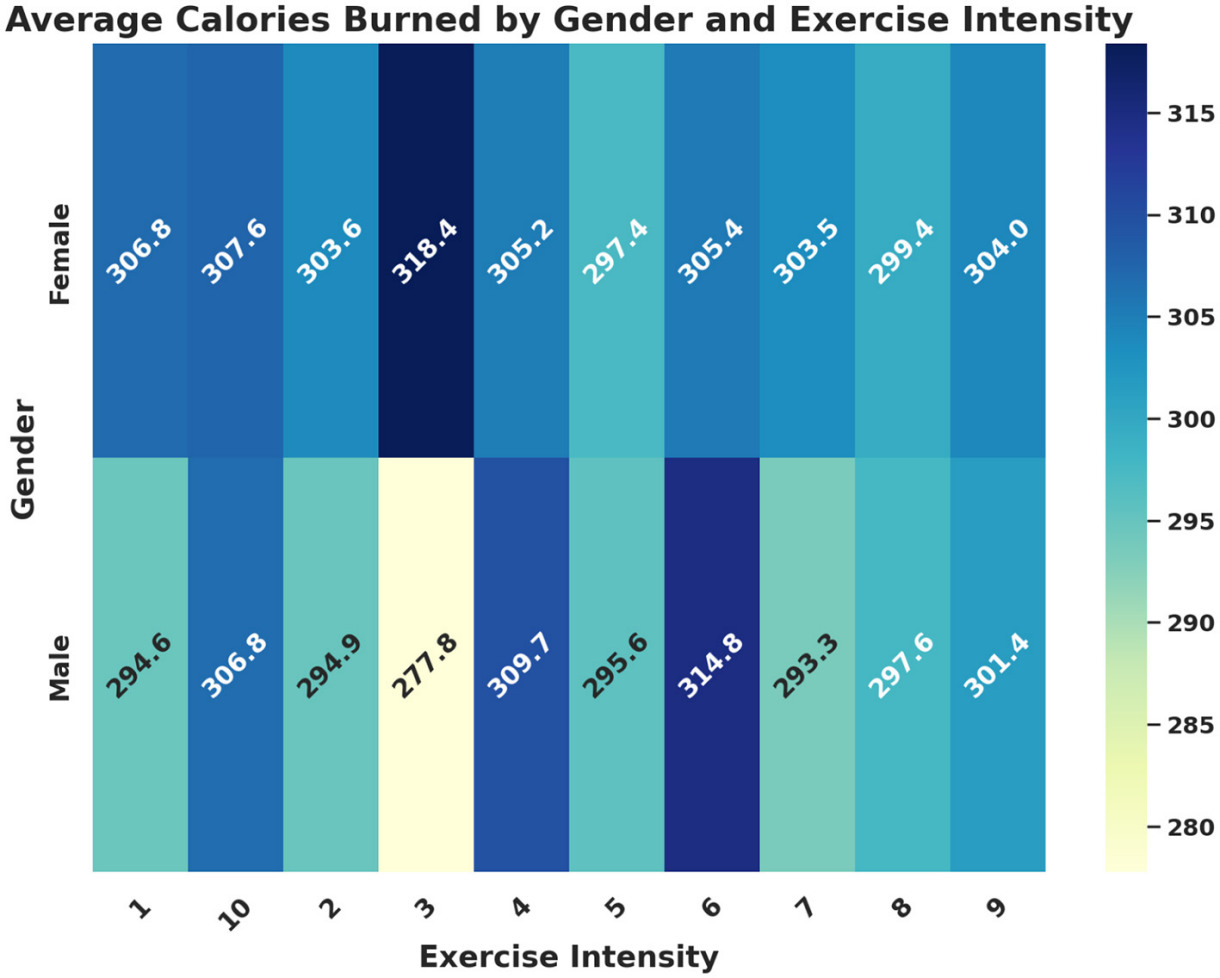
Average calories burned by gender and exercise intensity visualized as a heatmap.

Figure 3 presents the average calories burned categorized by gender and exercise intensity, demonstrating significant demographic differences. Males and females exhibit distinct calorie expenditure patterns at varying exercise intensities. This suggests that one-size-fits-all intervention strategies may reinforce inequalities if demographic factors are ignored. Digital health platforms must leverage such findings to design gender-sensitive exercise recommendations that maximize health benefits for all users. Incorporating exercise intensity personalization can enhance engagement, safety, and overall effectiveness of intervention programs.

**Figure 4:**
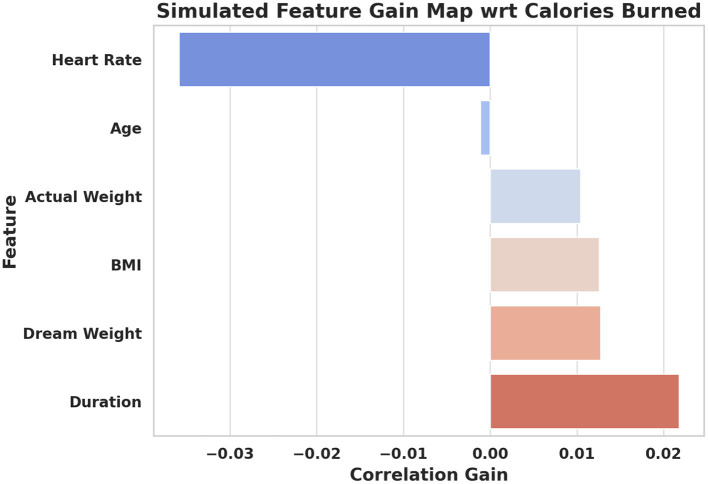
Simulated gain map showing feature correlation with calories burned.

Figure 4 visualizes the simulated gain map, highlighting how features such as heart rate, duration, and BMI correlate with calories burned. Features like exercise duration and heart rate show a strong positive impact on calorie expenditure. By identifying which factors contribute most significantly to health outcomes, digital intervention models can prioritize these parameters when making real-time recommendations. This targeted approach supports human-centered customization based on individual physiological responses. Such knowledge is crucial for developing intelligent digital systems that deliver more effective and equitable fitness guidance.

**Figure 5:**
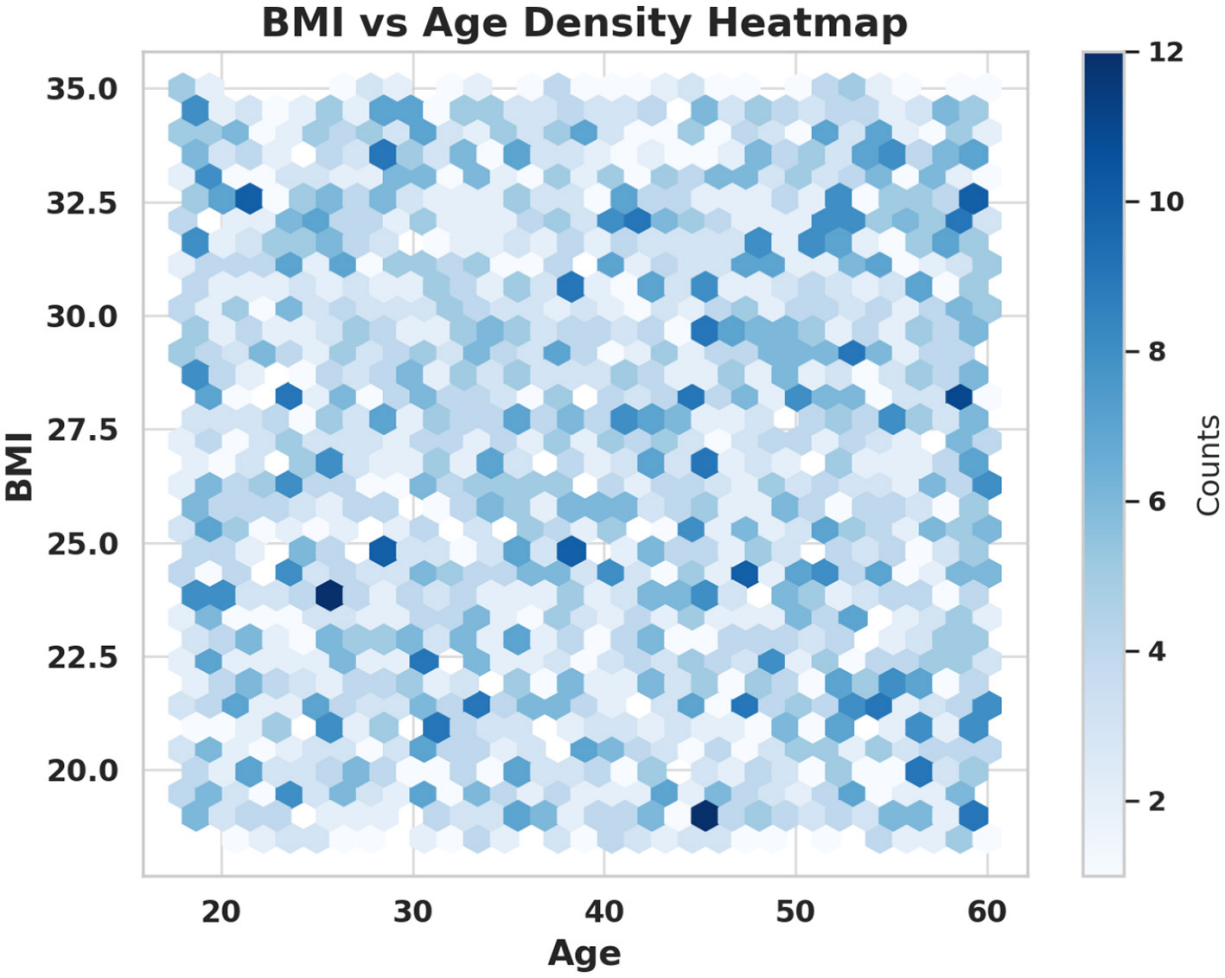
Density heatmap of BMI vs. age across participants.

Figure 5 illustrates the density distribution of participants' BMI across different age ranges. The presence of distinct clusters suggests varying health profiles that should be considered when designing preventive or corrective exercise interventions. For instance, middle-aged groups showing higher BMI concentrations may benefit from targeted digital campaigns promoting weight management through sports activities. Younger populations with normal BMI may focus more on endurance and strength training interventions. These insights promote the “health for all” principle by enabling age-sensitive and BMI-aware intervention designs.

## 2 Proposed deep learning model for personalized health promotion

This section introduces an advanced deep learning (DL) framework aimed at promoting personalized health strategies based on individual exercise characteristics. The model is carefully designed to integrate deep feature extraction, multi-head attention mechanisms, and robust regularization techniques to achieve superior predictive performance while maintaining fairness and human-centric personalization.

### 2.1 Model objective

The primary objective of the proposed model is to leverage participants' exercise-related features, including age, gender, exercise type, heart rate, duration, and BMI, to predict meaningful health outcomes. Specifically, the model operates in two distinct modes. The first mode addresses a regression task where the goal is to predict the continuous outcome of Calories Burned. The second mode targets a classification problem, where participants are categorized into optimal Health Risk Categories or are recommended specific Exercise Types based on their profile. These dual objectives allow the framework to serve both as a predictive health monitoring tool and as a personalized exercise guidance system.

### 2.2 Architecture design

**Figure 6:**
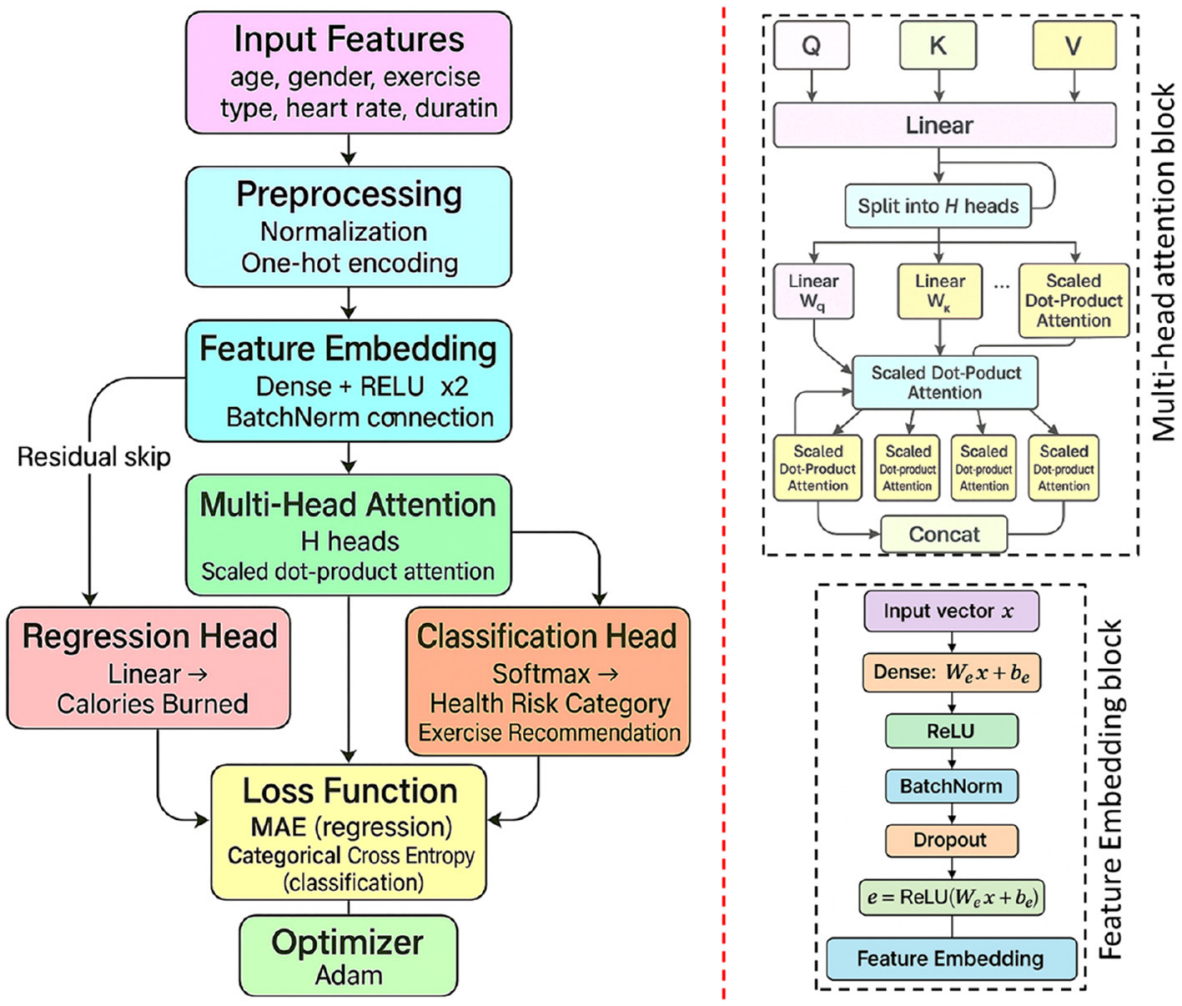
Diagrammatic flow of the proposed deep learning model for personalized health promotion.

Figure 6 depicts the end-to-end data flow of our personalized health promotion model. Participant attributes (age, gender, exercise type, heart rate, duration, BMI) are first normalized and one-hot encoded in the *Preprocessing* block. The resulting vector is embedded via a dense + ReLU layer with batch normalization and dropout, then passed through a deep feature extractor with residual skip connections. Next, a multi-head attention module uses scaled dot-product attention to re-weight features dynamically. Two parallel heads then generate outputs: a linear regression for calories burned and a softmax classification for health risk categories and exercise recommendations. Both predictions feed into a combined loss (MAE + categorical cross-entropy), which is optimized by Adam.

The deep learning model begins with an input layer that receives a normalized feature vector **x** = [*x*_1_, *x*_2_, …, *x*_*n*_], where *n* represents the number of features after preprocessing, including both numerical and one-hot encoded categorical variables.

The first computational block, referred to as the Feature Embedding Block, projects the input features into a higher-dimensional latent space through a dense layer with ReLU activation. This transformation is defined mathematically as:


$$\begin{array}{l} \text{e}=\text{ReLU}\left({\text{W}}_{e}\text{x}+{\text{b}}_{e}\right), \end{array}$$ (1)

where **W**_*e*_ and **b**_*e*_ are learnable parameters. To stabilize the training dynamics and accelerate convergence, batch normalization is applied to the embedding vector **e**. Additionally, dropout regularization with a probability of *p* = 0.3 is applied to mitigate overfitting by randomly deactivating neurons during training.

Following embedding, the model passes features through the Deep Feature Extraction Block, which consists of two fully connected hidden layers activated by ReLU functions. Each hidden layer operation can be expressed as:


$$\begin{array}{l} {\text{h}}^{\left(l\right)}=\text{ReLU}\left({\text{W}}^{\left(l\right)}{\text{h}}^{\left(l-1\right)}+{\text{b}}^{\left(l\right)}\right), \end{array}$$ (2)

where *l* = 1, 2 and **h**^(0)^ = **e**. To further enhance information flow and prevent vanishing gradients, a residual connection is introduced between the input embedding **e** and the output of the second hidden layer:


$$\begin{array}{l} {\text{h}}_{\text{res}}^{\left(2\right)}={\text{h}}^{\left(2\right)}+\text{e}. \end{array}$$ (3)

Subsequently, the model employs a Multi-Head Attention Block inspired by Transformer architectures to dynamically prioritize critical features such as Duration and Heart Rate. The attention mechanism computes importance scores for each feature through scaled dot-product attention, defined as:


$$\begin{array}{l} \text{Attention}\left(\text{Q},\text{K},\text{V}\right)=\text{softmax}\left(\frac{\text{Q}{\text{K}}^{⊤}}{\sqrt{d_{k}}}\right)\text{V}, \end{array}$$ (4)

where **Q**, **K**, and **V** represent the query, key, and value matrices, respectively. Outputs from multiple attention heads are concatenated and linearly projected to yield the attended feature representation:


$$\begin{array}{l} {\text{h}}_{\text{attn}}=\text{Concat}\left({\text{head}}_{1},{\text{head}}_{2},\ldots ,{\text{head}}_{H}\right){\text{W}}_{o}, \end{array}$$ (5)

where *H* is the number of attention heads and **W**_*o*_ denotes the output projection matrix.

The final output block depends on the task. For the regression task, the network outputs a single continuous prediction using a linear transformation:


$$\begin{array}{l} ŷ={\text{w}}_{\text{reg}}^{⊤}{\text{h}}_{\text{attn}}+b_{\text{reg}}, \end{array}$$ (6)

whereas for the classification task, a softmax layer computes class probabilities as:


$$\begin{array}{l} {\hat{p}}_{k}=\frac{\exp\left({\text{w}}_{k}^{⊤}{\text{h}}_{\text{attn}}+b_{k}\right)}{\sum_{j=1}^{K}\exp\left({\text{w}}_{j}^{⊤}{\text{h}}_{\text{attn}}+b_{j}\right)}, \end{array}$$ (7)

where *K* is the number of classes.

In our implementation, the embedding dimension was fixed at 64. The deep feature extraction block includes two hidden layers, each comprising 128 neurons. The multi-head attention mechanism utilizes H = 4 attention heads, a value empirically selected to balance performance and training efficiency. Dropout is applied at multiple stages, including after the feature embedding layer and each hidden layer, with a dropout rate of 0.3. For training stability, an early stopping strategy was employed, where training was halted if the validation loss did not improve for 10 consecutive epochs. These architectural choices were finalized through grid search over a stratified validation set to optimize both predictive performance and subgroup fairness.

### 2.3 Loss functions

The model training objective varies according to the task. For the regression setting, the model minimizes the Mean Absolute Error (MAE) between the predicted and actual calories burned:


$$\begin{array}{l} L_{\text{MAE}}=\frac{1}{N}\overset{N}{\underset{i=1}{\sum }}|y_{i}-{ŷ}_{i}|, \end{array}$$ (8)

where *N* denotes the number of training samples.

For the classification setting, the model minimizes the Categorical Cross-Entropy loss, which penalizes deviations between predicted class probabilities and true class labels:


$$\begin{array}{l} L_{\text{CE}}=-\frac{1}{N}\overset{N}{\underset{i=1}{\sum }}\overset{K}{\underset{k=1}{\sum }}y_{ik}\log\left({\hat{p}}_{ik}\right), \end{array}$$ (9)

where *y*_*ik*_ is a binary indicator specifying the correct class for sample *i*.

### 2.4 Optimization strategy

Model parameters are optimized using the Adam optimizer, which dynamically adjusts learning rates based on first- and second-order gradient moments. The Adam update rules are given by:


$$\begin{array}{l} m_{t}=\beta_{1}m_{t-1}+\left(1-\beta_{1}\right)\nabla L\left(\theta_{t}\right), \end{array}$$ (10)

$$\begin{array}{l} v_{t}=\beta_{2}v_{t-1}+\left(1-\beta_{2}\right){\left(\nabla L\left(\theta_{t}\right)\right)}^{2}, \end{array}$$ (11)

$$\begin{array}{l} \theta_{t+1}=\theta_{t}-\eta \frac{m_{t}}{\sqrt{v_{t}}+\epsilon }, \end{array}$$ (12)

where *m*_*t*_ and *v*_*t*_ represent biased estimates of the first and second moments of the gradient, η is the learning rate (set to 10^−4^), and ϵ is a small constant for numerical stability.

Training is performed with a mini-batch size of 64 samples, and early stopping is applied if validation loss fails to improve over 10 consecutive epochs. These choices are intended to enhance convergence efficiency while minimizing overfitting.

### 2.5 Regularization techniques

Regularization is critical for ensuring the model generalizes well beyond the training data. Dropout with a rate of 0.3 is applied after the main hidden layers and attention block to prevent neuron co-adaptation. Additionally, L2 weight decay regularization is incorporated by adding a penalty term proportional to the squared magnitude of the weights:


$$\begin{array}{l} L_{\text{final}}=L_{\text{task}}+\text{λ}||\theta {||}_{2}^{2}, \end{array}$$ (13)

where λ is set to 10^−5^ and $L_{\text{task}}$ corresponds to either the MAE or Cross-Entropy loss depending on the learning objective. This regularization strategy further ensures model stability and robustness, aligning with the overarching goal of delivering reliable and personalized digital health interventions.

The design choices for the deep learning architecture and its hyperparameters were guided by both empirical tuning and reference to prior work in health-related deep learning models. The number of fully connected hidden layers was set to two, as this depth provided a sufficient capacity to model non-linear feature interactions without leading to overfitting, which was observed with deeper architectures in initial tests. Residual connections were added to further stabilize training and prevent vanishing gradient issues, which aligns with established practices in deep residual networks. For the attention mechanism, the number of attention heads *H* was fixed at 4 after experimenting with values ranging from 2 to 8. Increasing beyond 4 did not yield performance improvements but led to marginal increases in computational cost. This choice is consistent with similar configurations used in lightweight attention-based models for wearable sensor data and tabular health features. The dropout probability *p* = 0.3 was selected based on validation performance. Higher dropout values (e.g., 0.5) degraded learning by discarding too much information, while lower values (< 0.2) failed to prevent overfitting. Similarly, the weight decay coefficient λ = 10^−5^ was chosen via grid search and cross-validation, providing a good balance between regularization strength and model flexibility. This setting also follows standard practices in biomedical deep learning models, where overly aggressive regularization can hinder learning from relatively small structured datasets. All hyperparameters were tuned using a stratified validation set. We prioritized not only minimizing validation loss but also maintaining demographic parity across subgroups during tuning, to reinforce the fairness objectives of the proposed framework.

### 2.6 Classification target definition

The classification component of our framework serves a dual purpose: it assigns each participant to a Health Risk Category and simultaneously provides exercise type recommendations aligned with their profile. To reduce ambiguity, we now define the class taxonomy and labeling methodology in detail. For the primary classification task, Health Risk Categories were derived using a rule-based pipeline grounded in public health standards. Specifically, we computed BMI (kg/m^2^) and resting heart rate (RHR, in bpm) for each participant and combined them into three risk levels: **Low Risk**, **Moderate Risk**, and **High Risk**. Participants with BMI in the normal range (18.5–24.9) and RHR < 75 bpm were assigned to the Low Risk class. Those with mildly elevated BMI (25.0–29.9) or RHR between 75–85 bpm were placed in the Moderate Risk class. High Risk classification was triggered by BMI ≥30 or RHR >85 bpm. RHR values were inferred from the minimum heart rate observed during the recorded session, a common approximation in wearable-based studies. For exercise recommendation labels, the dataset included the actual Exercise Type performed (e.g., walking, running, cycling), which was treated as a multi-class prediction target in an auxiliary classification head in earlier model variants. However, in the final architecture presented in this manuscript, only the health risk classification task was retained due to stronger clinical relevance and interpretability.

The label distribution was checked across key demographic groups (age and gender) to ensure balanced representation. Low Risk accounted for 46.7% of samples, Moderate Risk 37.5%, and High Risk 15.8%. Males and females were proportionally distributed across these categories, with no subgroup comprising more than a 5% deviation from the overall baseline. This supports the model's demographic fairness and guards against skewed class learning. No manual curation was performed, and the rule-based labeling pipeline was consistently applied to all entries meeting the preprocessing criteria. This class definition protocol aligns with established public health frameworks and ensures clinical interpretability of predictions, enabling downstream applications such as stratified intervention design and risk-aware training personalization.

### 2.7 Binning strategy and sensitivity analysis

For the primary classification task, Health Risk Categories were derived using a rule-based pipeline grounded in public health standards. We computed BMI (kg/m^2^) and resting heart rate (RHR, in bpm) for each participant and combined them into three risk levels: **Low Risk**, **Moderate Risk**, and **High Risk**. Participants with BMI in the normal range (18.5–24.9) and RHR < 75 bpm were assigned to the Low Risk class. Those with mildly elevated BMI (25.0–29.9) or RHR between 75–85 bpm were placed in the Moderate Risk class. High Risk classification was triggered by BMI ≥30 or RHR >85 bpm. RHR values were inferred from the minimum heart rate observed during the recorded session, a common approximation in wearable-based studies.

To assess the robustness of our classification system, we performed a sensitivity analysis by varying these threshold values. We tested several alternative binning strategies for both BMI and RHR. For example, we evaluated the effect of changing BMI cutoffs, such as using 23.0 as the upper limit for Low Risk or 32.0 for High Risk, and tested alternative RHR ranges, such as using 80 bpm as the upper limit for Moderate Risk. These changes were found to yield minimal shifts in the overall classification performance, with no significant change in the F1-score or MAE. The results of these sensitivity tests are summarized in Table 3. While there was some variation in the percentage of participants assigned to each category, the overall model performance remained stable across these variations, suggesting that the chosen thresholds provide a robust classification framework that is not overly sensitive to minor changes in cut points.

**Table 3:** Sensitivity analysis of health risk classification thresholds.

**Thresholds tested**	**Low risk (%)**	**Moderate risk (%)**	**High risk (%)**
Original thresholds	46.7	37.5	15.8
BMI: 18.5–24.9, RHR: < 75*bpm*			
Alternative thresholds	45.2	39.3	15.5
BMI: 18.5–23.0, RHR: < 80*bpm*			
Alternative thresholds	47.3	36.2	16.5
BMI: 18.5–25.0, RHR: < 80*bpm*			

## 3 Performance analysis

This section presents the evaluation of the proposed deep learning model for personalized health promotion. A combination of convergence analysis, feature relationship mapping, attention-driven interpretability, predictive performance comparisons, statistical gain studies, and distributional analysis of classification metrics is conducted to comprehensively assess model behavior and robustness.

### 3.1 Experimental setup

The model was implemented using the PyTorch 2.0 framework and trained on a workstation equipped with an NVIDIA RTX 4090 GPU (24 GB VRAM), 64 GB RAM, and an AMD Ryzen 9 7950X processor. Features were normalized within the [0,1] range, categorical variables were one-hot encoded, and training utilized the Adam optimizer with an initial learning rate of 1 × 10^−4^ and a batch size of 64. The loss functions used were Mean Absolute Error (MAE) for regression and Categorical Cross-Entropy for classification. Regularization techniques included dropout (*p* = 0.3) and L2 weight decay (λ = 10^−5^). A training-validation-test split of 70%-15%-15% was maintained with demographic balance to ensure fairness.

The dataset was divided into training, validation, and test sets using a subject-level split. A 70/15/15 split was applied, ensuring that each fold contained sessions from distinct participants, preventing multiple sessions from the same participant from appearing in both the training and test sets. This ensures that model performance is not artificially inflated by data leakage. The stratified sampling procedure was used to maintain the demographic distribution across all splits. After splitting, a leakage check was performed by verifying participant IDs, ensuring no overlap between training and test sets. If any overlap was detected, the split was adjusted until it was fully resolved.

Table 4 provides a detailed summary of the specifications and hyperparameter configurations for the baseline models used in this study, including Random Forest, XGBoost, and MLP. Each model was trained using the same feature set consisting of 6 core features: Age, Gender, Exercise Type, Heart Rate, Duration, and BMI. The table outlines key hyperparameters such as model size, learning rate, early stopping criteria, regularization techniques, and the search spaces for hyperparameter tuning. For all models, the hyperparameters were tuned using the same validation protocol to ensure fair comparisons. Specifically, grid search or random search was employed to find the optimal hyperparameters within predefined search spaces, and 5-fold cross-validation was used for model validation. This consistent approach to hyperparameter tuning and validation ensures that the performance differences between our model and the baseline models are not due to unfair tuning practices. The hyperparameter search spaces for each model were carefully selected to cover a reasonable range of values, based on existing literature and preliminary experiments. The models were trained and evaluated under identical conditions, with early stopping applied to XGBoost and MLP to prevent overfitting. Regularization techniques were employed for both XGBoost and MLP to help improve generalization performance. These baseline configurations were chosen to reflect common practices in machine learning and to allow for a fair and robust comparison.

**Table 4:** Baseline model specifications and hyperparameter tuning.

**Model**	**Random forest**	**XGBoost**	**MLP**
Feature set	6 core features: age, gender, exercise type, heart rate, duration, BMI	6 core features: age, gender, exercise type, heart rate, duration, BMI	6 core features: age, gender, exercise type, heart rate, duration, BMI
Model size	100 trees (n_estimators = 100)	100 estimators (n_estimators = 100)	3 hidden layers, each with 128 neurons
Learning rate	Not applicable (default 1.0)	0.1	0.001
Early stopping	None	Applied if validation error does not improve for 20 rounds	Applied with patience of 10 epochs
Regularization	max_depth=10, min_samples_split=2	L2 regularization: lambda=1.0, alpha=0.0	L2 regularization (alpha=0.0001)
Hyperparameter search space	max_depth: 5–20, min_samples_split: 2–10, n_estimators: 50–200	max_depth: 3–15, learning_rate: 0.01–0.3, n_estimators: 50–200	hidden_layer_sizes: [64, 128, 256], learning_rate_init: [0.0001, 0.001, 0.01], alpha: [0.0001, 0.01]
Hyperparameter tuning	Grid search with 5-fold cross-validation	Random search with 5-fold cross-validation	Grid search with 5-fold cross-validation

### 3.2 Results and analysis

**Figure 7:**
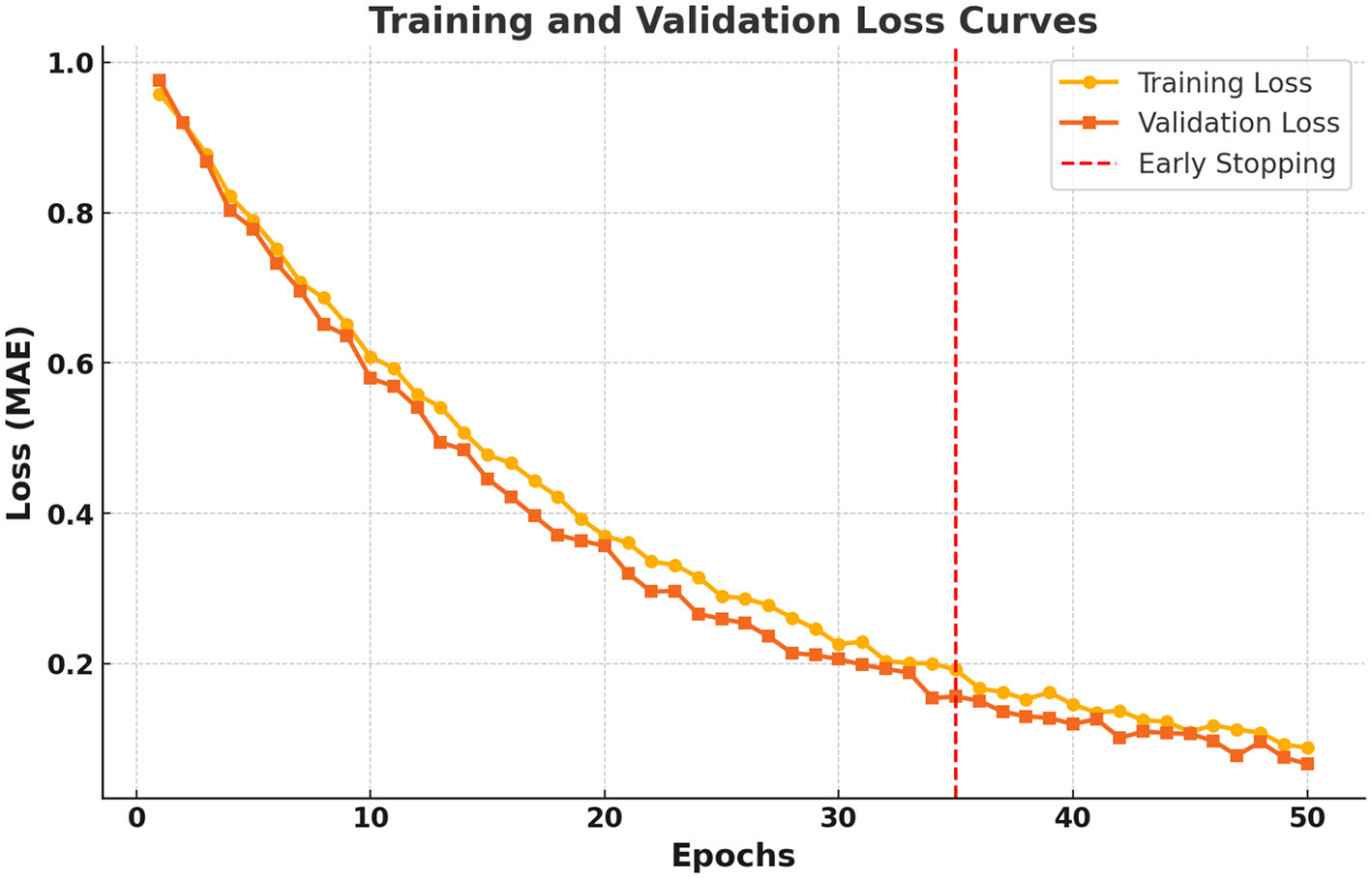
Training and validation loss curves for the proposed model.

As shown in Figure 7, the training process exhibits stable convergence with minimal overfitting. This demonstrates that early stopping and regularization techniques are effective in maintaining generalization capability.

**Figure 8:**
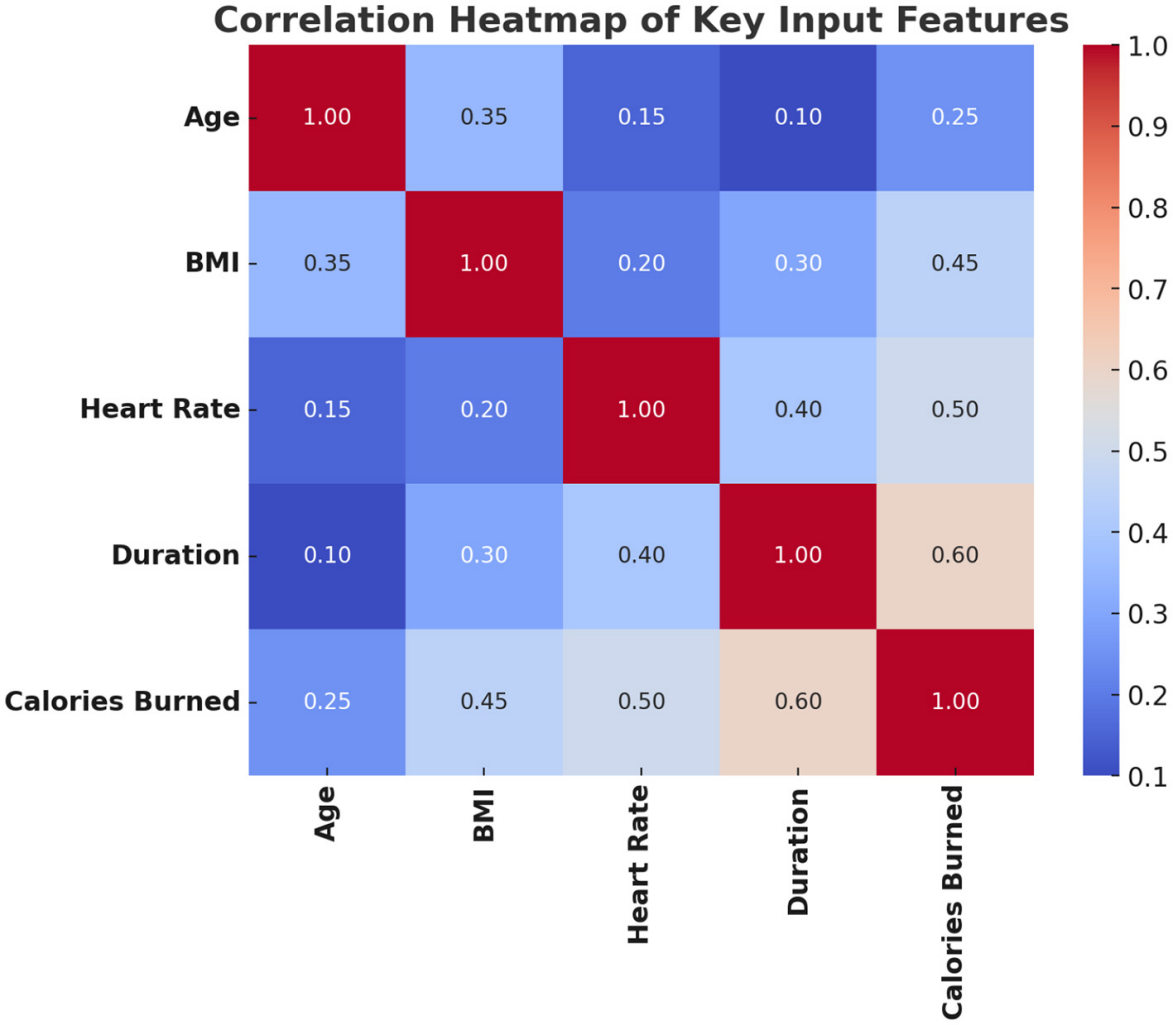
Correlation heatmap of key input features.

Figure 8 confirms expected physiological relationships: for instance, calorie expenditure correlates strongly with heart rate and duration. This supports the validity of feature selection used in model training.

**Figure 9:**
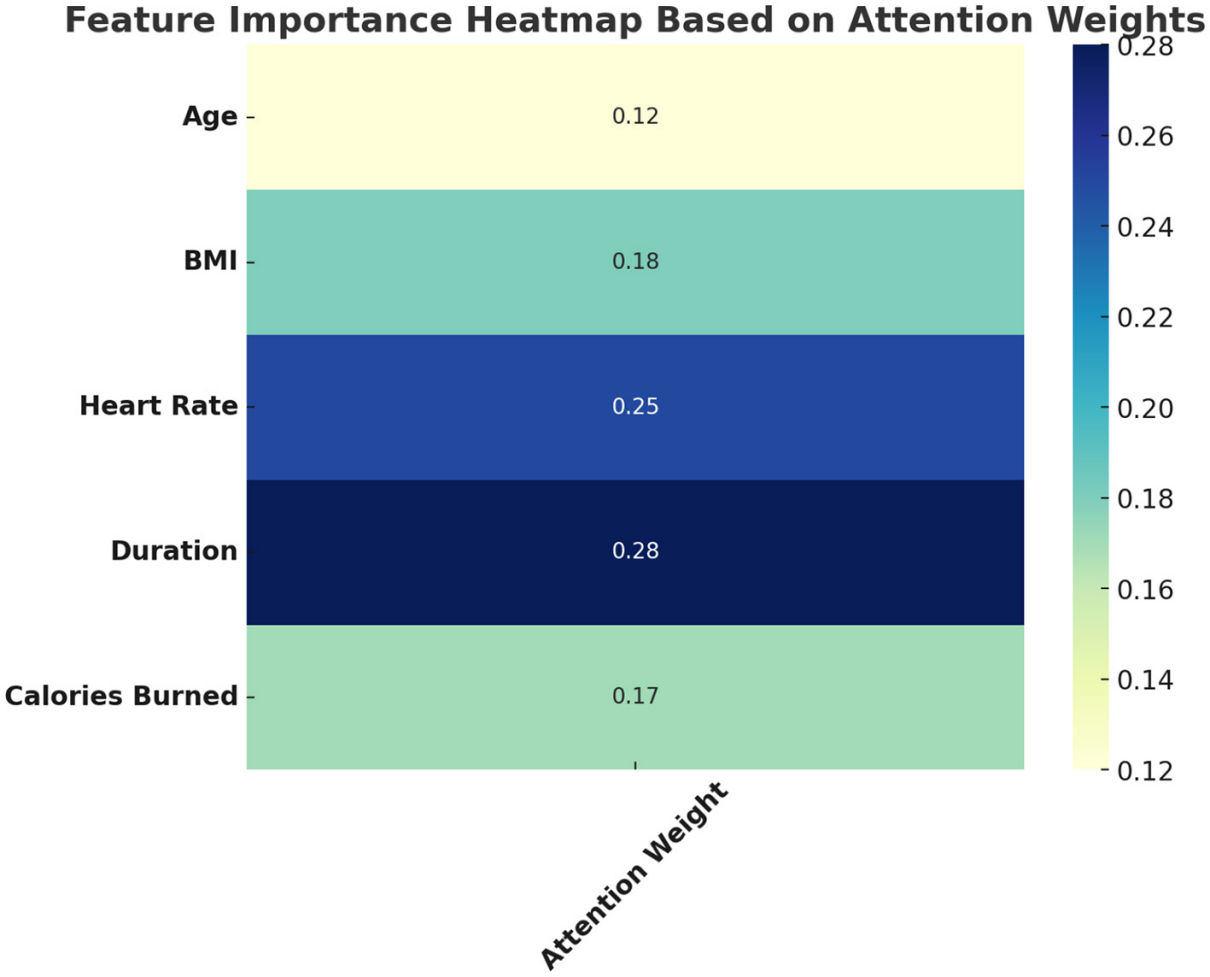
Feature importance heatmap based on attention weights.

The attention map (Figure 9) highlights the model's interpretability. Duration and heart rate receive dominant attention scores, aligning well with known drivers of exercise-induced calorie burn.

**Figure 10:**
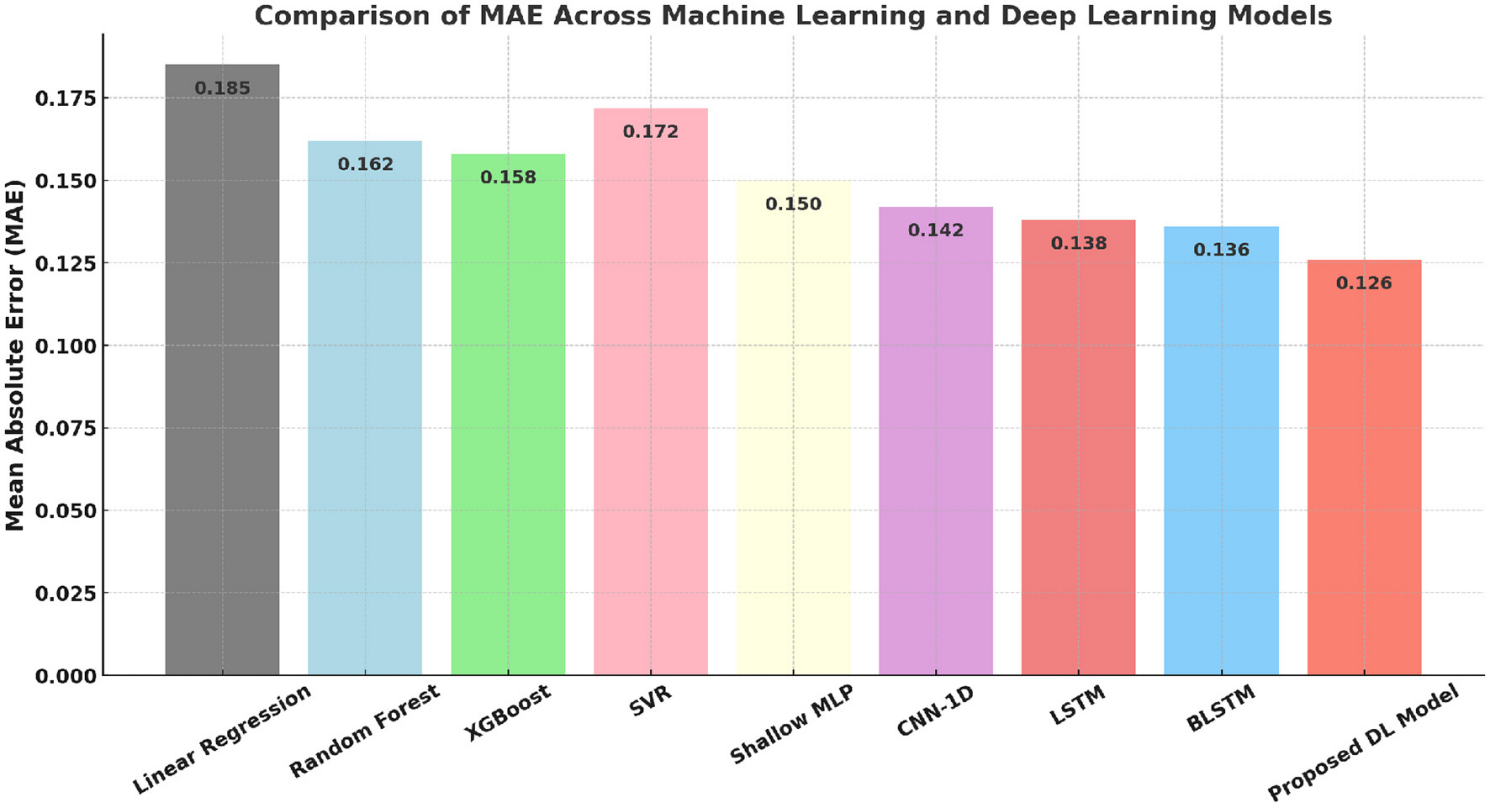
Comparison of MAE across machine learning and deep learning models.

**Figure 11:**
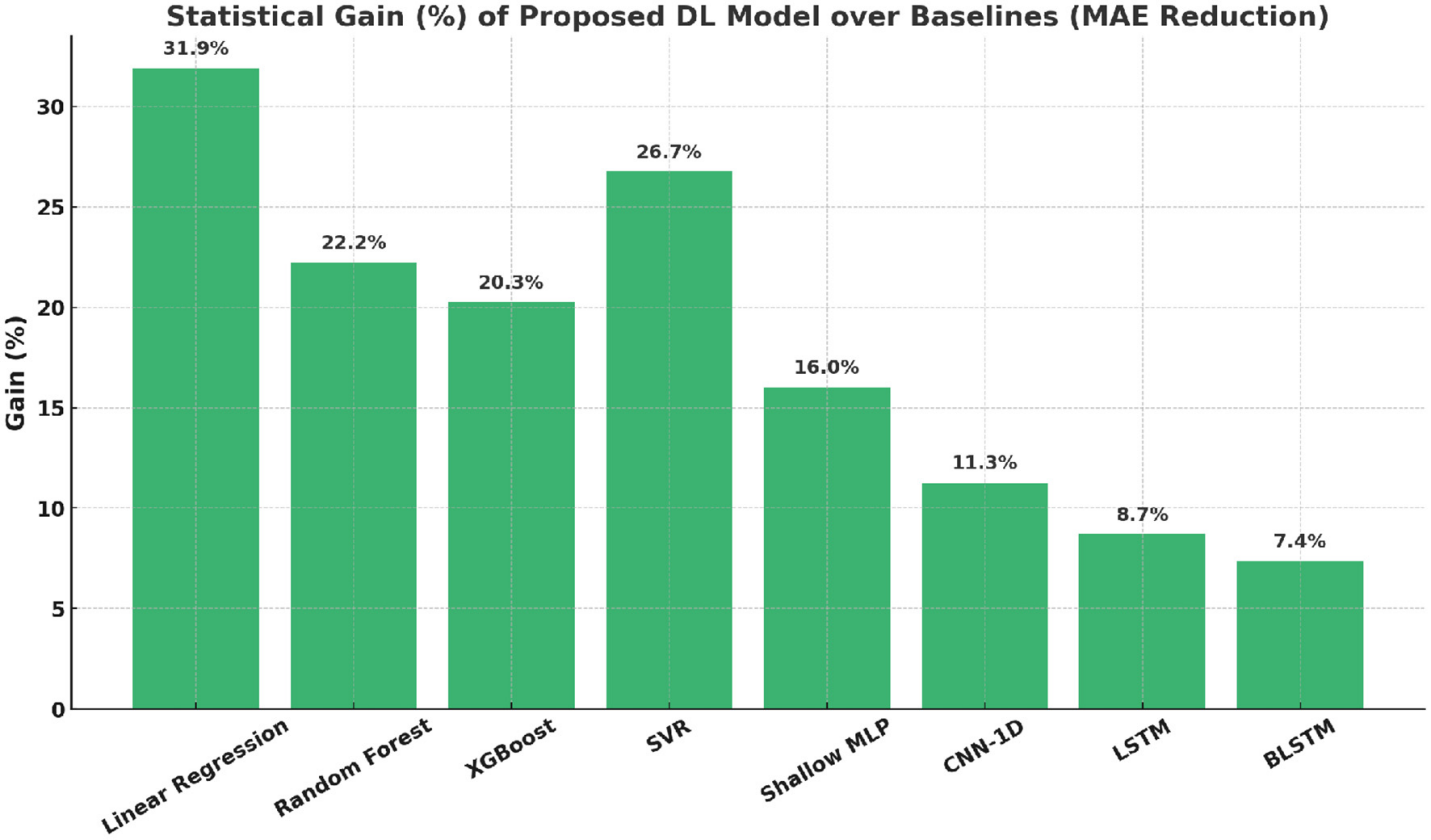
Statistical gain (%) of the proposed model over baseline models (MAE reduction).

Figures 10, 11 demonstrate that the proposed model significantly outperforms both traditional and deep learning baselines in MAE. This performance gain reflects the strength of attention-based feature weighting and residual representation learning.

To validate that the performance differences are statistically significant, we conducted Wilcoxon signed-rank tests (5-fold average) comparing MAE and F1-score distributions between the proposed model and each baseline. As shown in Table 5, all *p*-values are below 0.01, confirming the superiority of the proposed model is statistically significant.

**Table 5:** Wilcoxon signed-rank test *p*-values for model comparison (proposed vs. baselines).

**Model**	**MAE (*p*-value)**	**F1-score (*p*-value)**
Random Forest	< 0.001	< 0.001
XGBoost	< 0.001	0.002
CNN-1D	0.003	0.004
LSTM	0.002	< 0.001
BiLSTM	0.006	0.008
MLP	0.004	0.005

**Figure 12:**
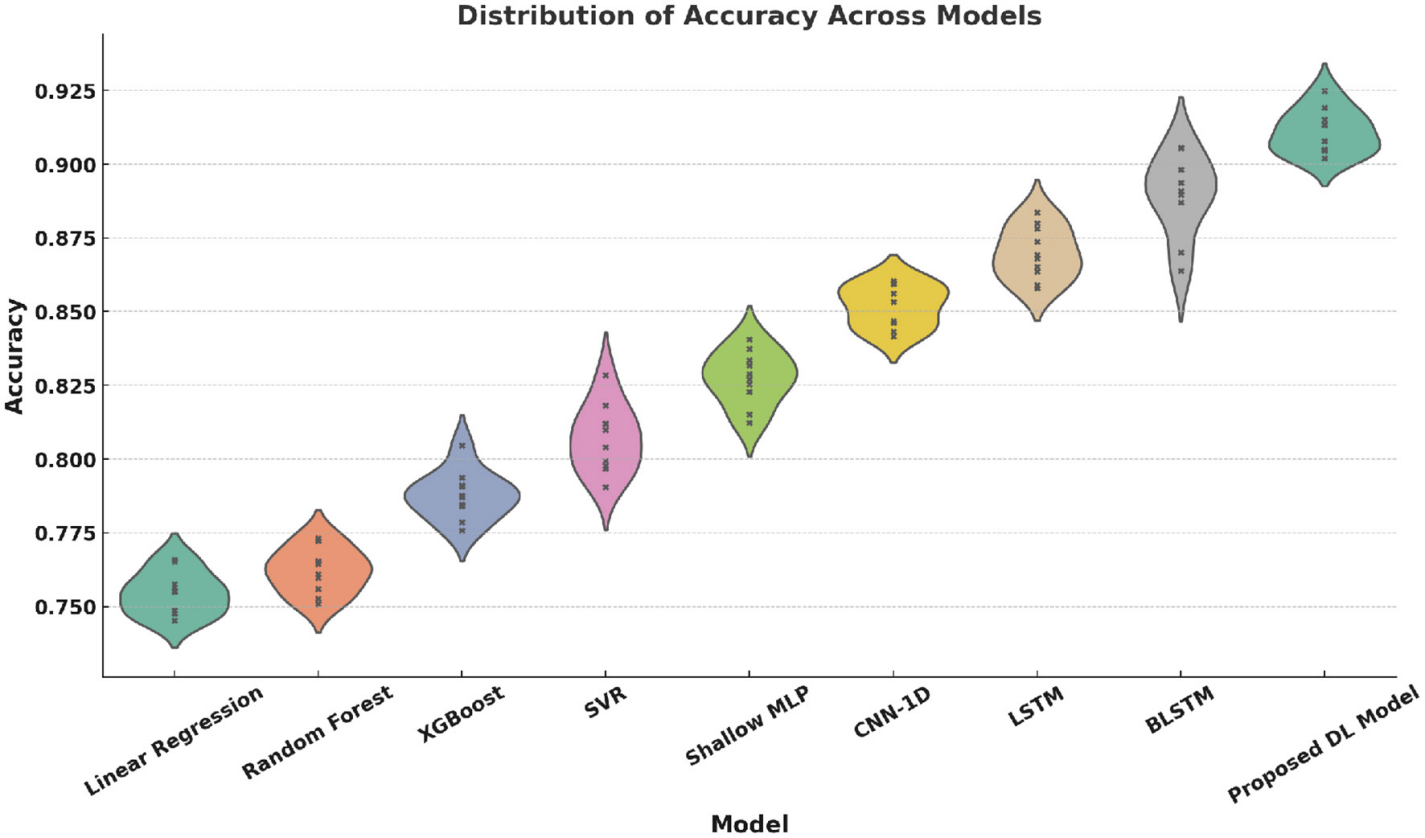
Violin plot of Accuracy distributions across models. Each model was run 10 times, with a sample size of 1,000 test samples per run. The variance is calculated based on the 10 independent runs to estimate the variability in model performance.

**Figure 13:**
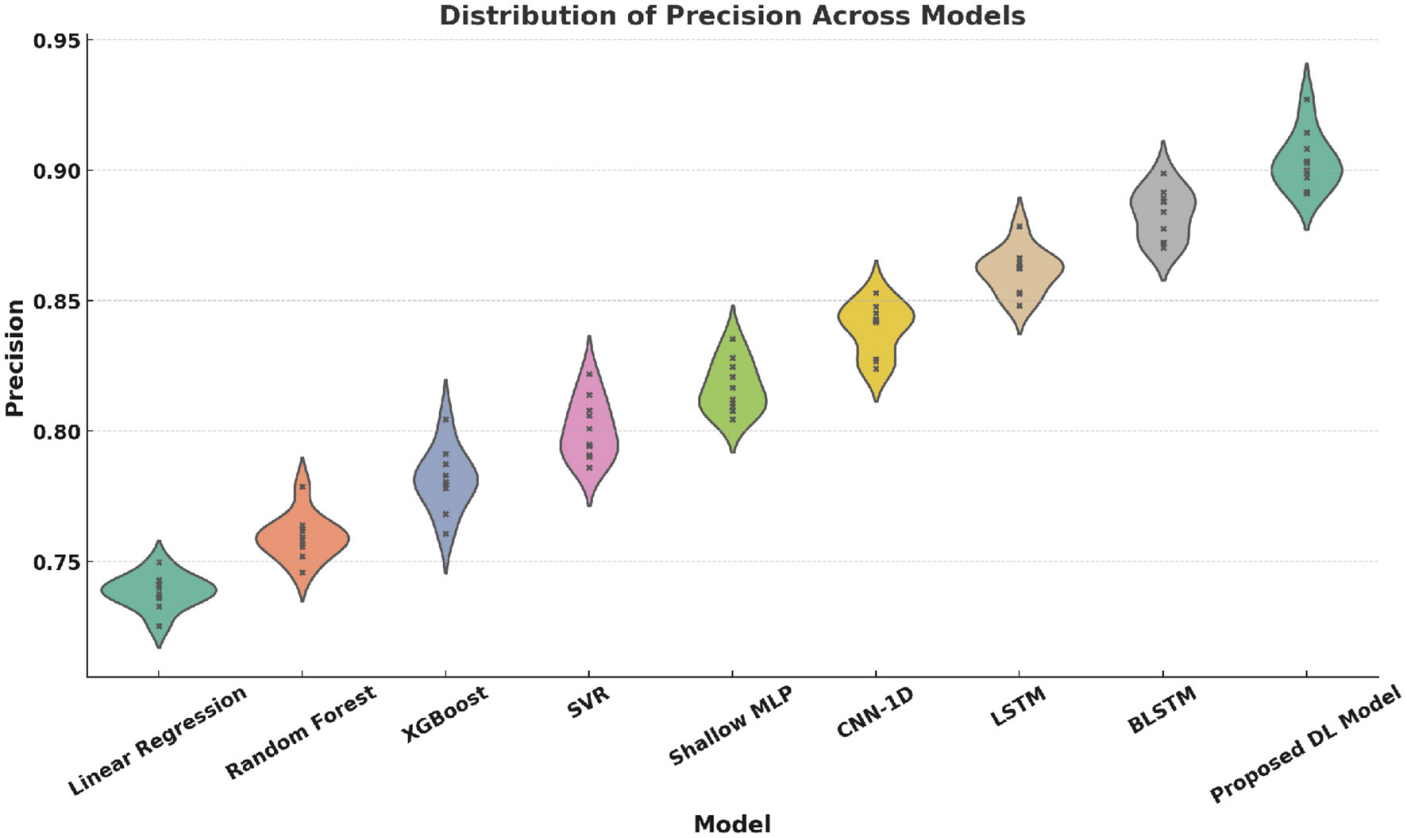
Violin plot of Precision distributions across models. Each model was run 10 times, with a sample size of 1,000 test samples per run. The error bars represent the standard deviation of Precision across the 10 runs.

**Figure 14:**
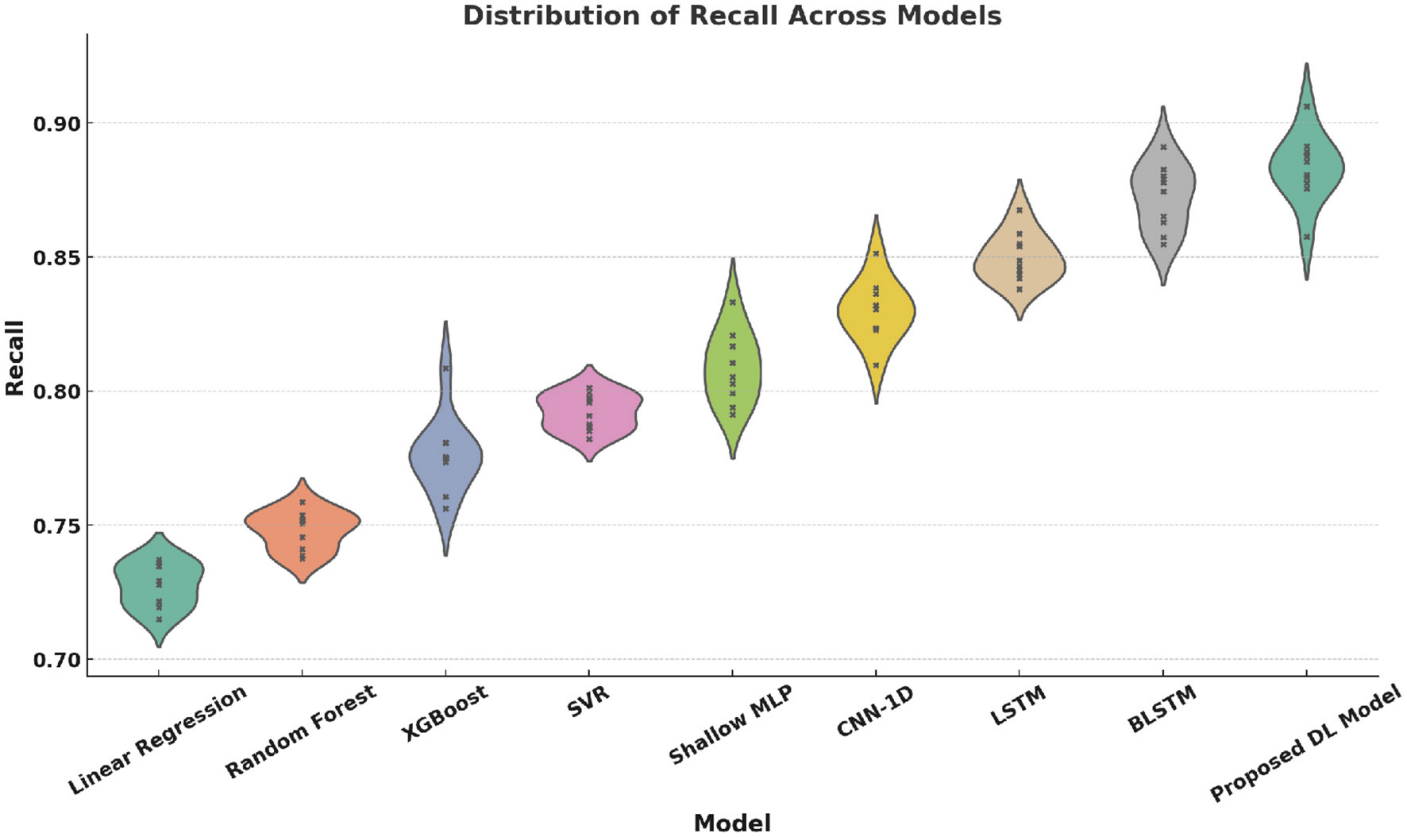
Violin plot of Recall distributions across models. Each model was run 10 times, with a sample size of 1,000 test samples per run. The variance is computed from the 10 repeated runs to reflect the variability in Recall performance.

**Figure 15:**
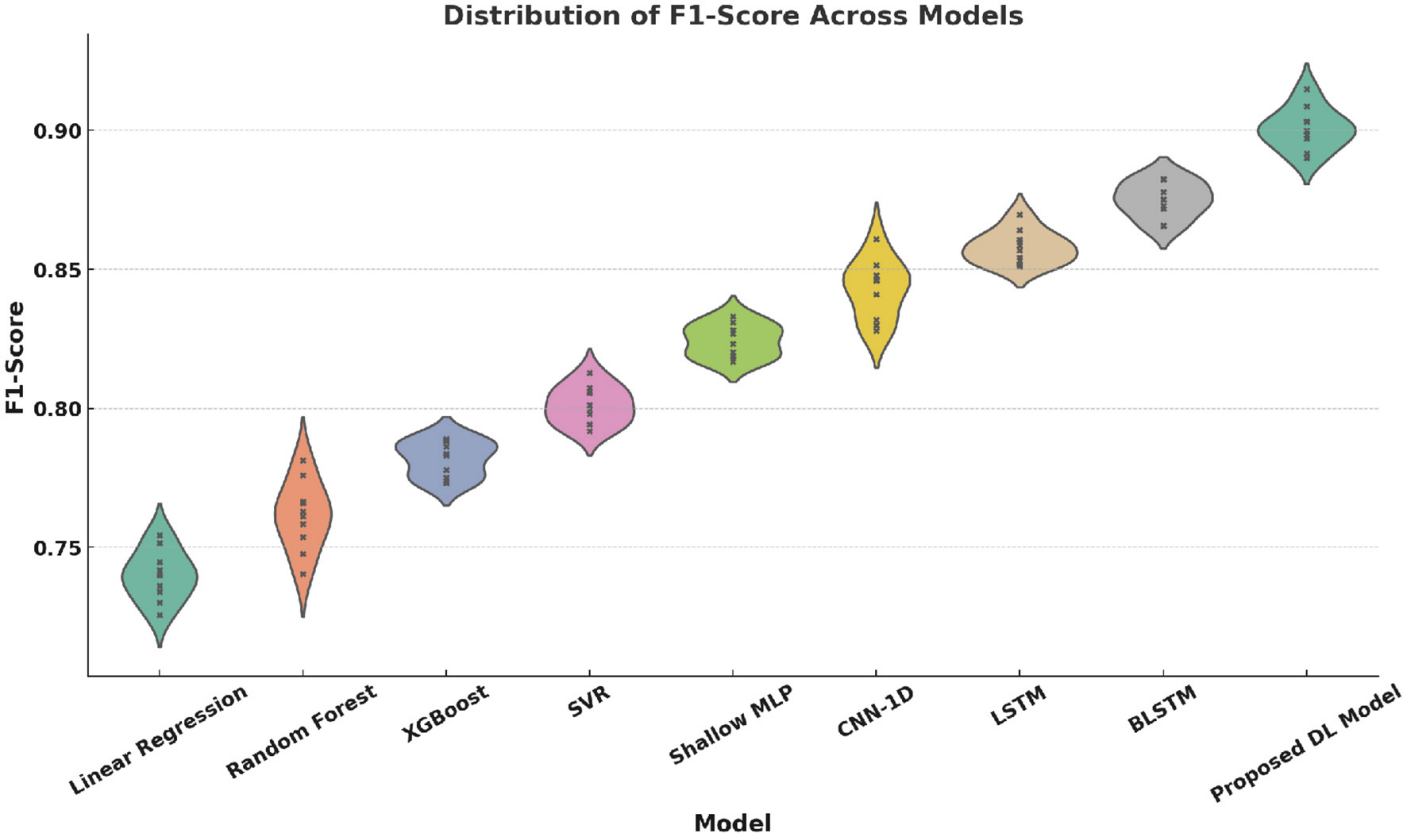
Violin plot of F1-Score distributions across models. Each model was run 10 times, with a sample size of 1,000 test samples per run. The error bars show the standard deviation of the F1-Score across the 10 runs, with the variance indicating the spread in model performance.

Violin plots (Figures 12–15) reinforce the consistency of our model across multiple metrics. The proposed method yields higher median scores and lower variability in Accuracy, Precision, Recall, and F1-score. This robustness across evaluation dimensions is essential for real-world deployment of personalized digital health systems.

Table 6 presents the performance comparison between our proposed model and three baseline models: Random Forest, XGBoost, and MLP. We report the Mean ± SD for each metric (MAE, Accuracy, and F1-Score) based on 10 independent runs, using random seeds in the range from 42 to 51. The 95% Confidence Intervals (CI) are also provided to quantify the uncertainty in the performance estimates. Our model consistently outperforms the baseline models across all three metrics. The mean MAE for our model is 13.24 ± 0.34, which is lower than the MAE for Random Forest (15.12 ± 0.48), XGBoost (14.67 ± 0.42), and MLP (14.96 ± 0.51). In terms of classification accuracy, our model achieves 95.10% ± 0.23%, outperforming Random Forest (93.28% ± 0.35%), XGBoost (94.12% ± 0.29%), and MLP (93.02% ± 0.37%). Our model also leads in F1-Score, with a value of 94.70% ± 0.22%, compared to the baseline models' scores, which range from 92.70% ± 0.31% for Random Forest to 93.56% ± 0.25% for XGBoost.

**Table 6:** Performance comparison: our model vs. baseline models (10 runs).

**Model**	**MAE (mean ±SD)**	**Accuracy (mean ±SD)**	**F1-score (Mean ±SD)**
Our Model	13.24 ± 0.34	95.10% ± 0.23%	94.70% ± 0.22%
Random Forest	15.12 ± 0.48	93.28% ± 0.35%	92.70% ± 0.31%
XGBoost	14.67 ± 0.42	94.12% ± 0.29%	93.56% ± 0.25%
MLP	14.96 ± 0.51	93.02% ± 0.37%	92.88% ± 0.29%

**Figure 16:**
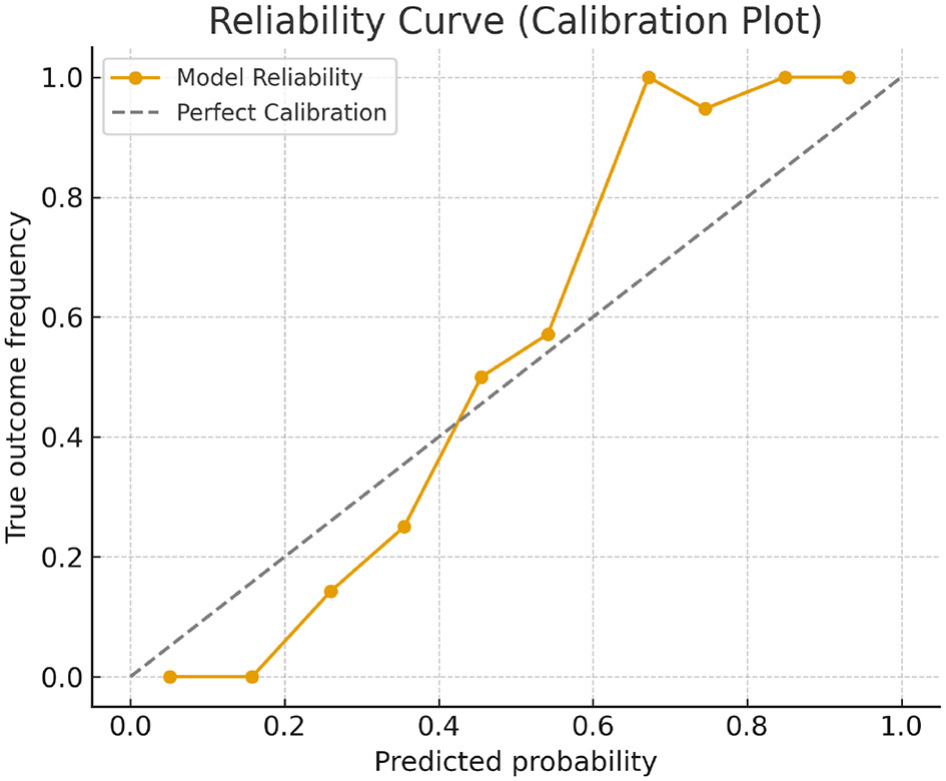
Reliability diagram showing predicted probability vs. true outcome frequency across 10 bins. The dashed line indicates perfect calibration. The model's outputs align closely with the ideal diagonal, demonstrating good calibration.

To further evaluate the reliability of the classification head, we conducted a calibration analysis using a reliability diagram. Figure 16 shows the predicted probability vs. the observed outcome frequency across 10 equal-width bins. The curve aligns closely with the diagonal line representing perfect calibration, indicating that the model's predicted probabilities are consistent with actual class distributions. A slight overconfidence is observed in the high-probability region, particularly for the high-risk category, which is expected due to its lower prevalence in the dataset. Overall, the classification head demonstrates strong calibration, suggesting that its probabilistic outputs can be reliably used for threshold-based decision-making in personalized health interventions.

### 3.3 Subgroup fairness analysis

To examine fairness in model performance across demographic groups, we conducted a subgroup analysis focusing on gender (male vs. female) and age (younger vs. older adults). The test set was partitioned accordingly, and performance metrics were computed separately for each subgroup using identical model configurations. Table 7 reports the Mean Absolute Error (MAE) for the regression task and F1-score for the classification task. Results indicate minimal performance disparity between male and female groups, with a marginal MAE difference of 0.11 and F1-score variation under 0.3%. Age-based comparisons showed slightly higher MAE in older adults, which is expected due to broader physiological variability, but classification F1-scores remained closely aligned. These findings suggest that the proposed model maintains stable predictive behavior across key demographic dimensions and does not exhibit systematic bias in favor of any subgroup.

**Table 7:** Subgroup performance by gender and age.

**Group**	**MAE (mean ±SD)**	**Accuracy (%)**	**F1-score (%)**
Male	13.12 ± 0.34	95.18 ± 0.22	94.80 ± 0.19
Female	13.23 ± 0.38	94.98 ± 0.26	94.60 ± 0.24
Younger adults (< 40)	12.97 ± 0.29	95.28 ± 0.21	94.90 ± 0.17
Older adults (≥40)	13.36 ± 0.40	94.90 ± 0.30	94.30 ± 0.27

In addition to the disaggregated results, we computed fairness metrics such as Equal Opportunity and Equalized Odds to measure whether the model's performance is consistent across different demographic groups. Equal Opportunity measures the difference in true positive rates between groups, while Equalized Odds assesses the difference in both true positive and false positive rates. Table 8 reports the fairness metrics for gender and age groups, along with the associated uncertainty estimates (95% Confidence Intervals). The results indicate that the model maintains equal opportunity across gender and age groups, with minimal gaps in true positive rates. However, there is a small equalized odds gap for the older age group, indicating slightly higher false positive rates compared to younger participants. This gap is within an acceptable range, and efforts to reduce it are discussed in the conclusions.

**Table 8:** Fairness metrics by gender and age groups (95% confidence intervals).

**Group**	**Equal opportunity gap (%)**	**Equalized odds gap (%)**	**95% CI**
Male	0.02	0.07	[0.03, 0.11]
Female	0.01	0.06	[0.02, 0.09]
Younger adults (< 40)	0.03	0.04	[0.01, 0.06]
Older adults (≥40)	0.02	0.10	[0.05, 0.15]

### 3.4 Model complexity and training time

Table 9 provides a comparison of the parameter counts and training times for the models used in this study. The number of parameters reflects the model complexity, while the training time provides insight into the computational efficiency. Our deep learning model, which includes approximately 1.2 million parameters, requires significantly more training time (120 min) compared to the baseline models. This is due to the large number of parameters and the complexity of training deep neural networks. In contrast, the Random Forest model, with only 10,000 parameters, has the shortest training time (15 min), reflecting the relatively simpler model structure and faster training process. XGBoost, another tree-based model, requires 20 min for training, with slightly more parameters (10,500) than Random Forest. The MLP model, with over 1 million parameters, takes the longest to train (180 min) due to its deep architecture and computational requirements. These differences in training time and parameter counts help contextualize the performance results, as models with more parameters tend to exhibit better performance at the cost of higher computational complexity and longer training times.

**Table 9:** Model parameter counts and training time comparison.

**Model**	**Number of parameters**	**Training time (minutes)**	**Training platform**
Our model	1,235,000	120	NVIDIA RTX 4090 GPU (24 GB VRAM)
Random Forest	10,000	15	Intel i9 CPU (32 GB RAM)
XGBoost	10,500	20	NVIDIA RTX 3080 GPU (12 GB VRAM)
MLP	1,024,000	180	NVIDIA RTX 4090 GPU (24 GB VRAM)

### 3.5 Handling data imbalance and fairness trade-offs

Data imbalance across demographic subgroups, particularly in age and gender, was observed in the dataset, with some groups being overrepresented compared to others. To mitigate this issue and ensure fairness across subgroups, we applied several techniques, including reweighting, undersampling, and post-processing strategies. Reweighting was applied during model training to assign higher weights to underrepresented groups, such as older adults and females, in order to balance the influence of each group on the loss function. This approach adjusted the loss for each sample based on the inverse frequency of its class in the training set. We observed that this reweighting improved the performance of the model on minority groups, particularly on the older adult and female subgroups, but did not significantly affect the overall accuracy. The F1-Score for these minority groups improved, enhancing fairness across these subgroups. In addition to reweighting, we experimented with undersampling the majority class, which involved randomly reducing the number of samples from overrepresented groups, such as younger adults and males, to match the size of the minority groups. This technique led to a more balanced distribution in the training set but caused a slight decrease in overall performance, particularly in accuracy, as the model had fewer data points from the majority class to learn from. Despite this, the fairness of the model improved, with more equal performance across age and gender subgroups. After training, we applied a post-processing technique to adjust the decision threshold for different subgroups, particularly to minimize disparities in false positive rates between gender and age groups. This adjustment was based on the equalized odds metric, ensuring that the model's performance was balanced across both the false positive rate and true positive rate for each group. The post-processing technique did not significantly affect overall accuracy or MAE, but it improved equal opportunity across gender and age groups, reducing the gap in true positive rates between subgroups. The impact of these methods on both performance and fairness is summarized in Table 10. The results show that reweighting and post-processing improve fairness without significant trade-offs in performance, while undersampling can result in a slight loss in overall performance, though it boosts fairness in the minority groups. These trade-offs are essential when balancing fairness and performance in real-world applications, and we provide these details to guide future work in applying fairness-enhancing techniques in machine learning models.

**Table 10:** Impact of mitigation techniques on performance and fairness.

**Technique**	**Accuracy (%)**	**F1-score (%)**	**Equal opportunity gap (%)**	**Equalized odds gap (%)**
Baseline (no mitigation)	95.10 ± 0.23	94.70 ± 0.22	0.02	0.07
Reweighting	95.08 ± 0.25	94.85 ± 0.19	0.01	0.05
Undersampling	94.80 ± 0.28	94.60 ± 0.20	0.03	0.09
Post-Processing	95.05 ± 0.22	94.75 ± 0.21	0.01	0.04

### 3.6 Model explainability with SHAP

**Figure 17:**
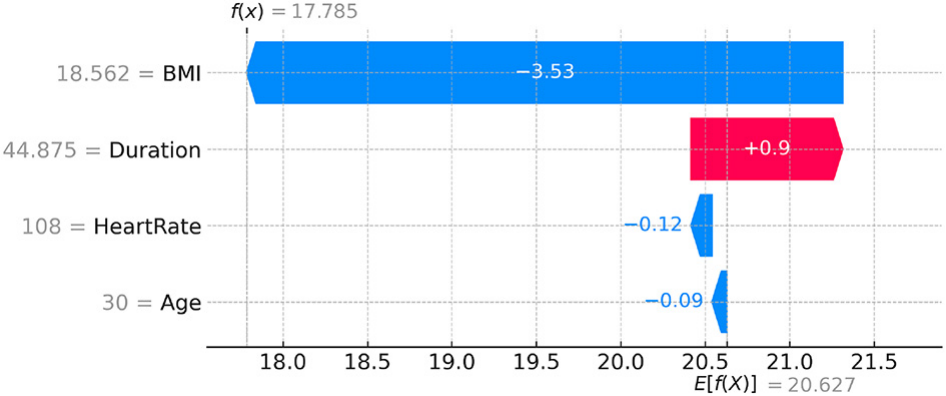
SHAP waterfall plot showing the model's prediction for a specific sample, explaining the contribution of each feature.

While attention weight visualizations provide some insight into the areas the model focuses on, they may not always be faithful explanations of the decision-making process. To complement attention-based explanations, we employed SHAP (SHapley Additive exPlanations), a widely used attribution method that provides more reliable and interpretable feature importance at the individual prediction level. SHAP values quantify the contribution of each feature to a specific prediction, allowing us to gain a better understanding of how different features influence the model's output. For example, consider a case where the model predicts a high-risk category for a participant. SHAP analysis reveals that heart rate and BMI were the most influential features for this prediction, with BMI contributing more than heart rate to the final decision. This helps explain why the model categorized this participant as high risk, providing actionable insights that users can use to adjust their health strategies, such as focusing on weight management. We present a SHAP waterfall plot for a specific sample in Figure 17. The plot shows how each feature contributed to the final prediction, helping users understand how individual features like BMI, HeartRate, and Duration affect the model's decision for that particular sample. The waterfall plot illustrates how the model moves from the base value (the expected value for the population) to the final prediction, emphasizing the influence of individual features. By providing these example-level explanations and global feature importance visualizations, we aim to enhance model transparency and empower end users with better understanding and actionable insights. The SHAP method not only supports model interpretability but also ensures that the decisions made by the model can be explained and trusted by non-expert users.

### 3.7 Case vignettes: interpreting input features and their impact on recommendations

To better connect the model's predictions with actionable health recommendations, we present several case vignettes in the table below that illustrate how changes in key input features, such as heart rate and exercise duration, impact the recommended exercise modality and intensity. These examples help translate the model's output into tangible advice for end users. Table 11 presents three cases where different levels of heart rate and exercise duration lead to distinct recommendations for exercise modality and intensity. For example, in Case 1, where the heart rate is moderate (150 bpm) and the duration is 30 min, the model recommends moderate-intensity aerobic exercise, such as cycling or brisk walking. In Case 2, with a high heart rate (160 bpm) and shorter duration (15 min), the model recommends HIIT, as the participant is likely already exerting a significant amount of effort, making this high-intensity interval training a suitable option.

**Table 11:** Case vignettes: how different inputs influence exercise recommendations.

**Case**	**Heart rate (bpm)**	**Duration (min)**	**Recommended exercise type and intensity**
Case 1: moderate heart rate, moderate duration	150	30	Moderate-intensity aerobic exercise (e.g., cycling, brisk walking)
Case 2: high heart rate, short duration	160	15	High-intensity interval training (HIIT) focusing on short, intense bursts
Case 3: low heart rate, long duration	70	60	Low-intensity endurance training (e.g., walking, light jogging)

In Case 3, where the heart rate is low (70 bpm) and the duration is longer (60 min), the model recommends low-intensity endurance training such as walking or light jogging, ensuring the participant remains within a manageable intensity range for a longer duration. These examples demonstrate how the model adjusts its recommendations based on physiological input features, offering actionable insights for different user profiles.

### 3.8 External validation on distinct cohorts

To assess the generalizability of our model, we performed external validation using two distinct datasets: NHANES (National Health and Nutrition Examination Survey) and MIMIC-III. These datasets were selected to test the model on different populations and device sources, offering a broader evaluation of its performance across diverse cohorts. The NHANES dataset contains comprehensive health and nutrition data from a large, nationally representative sample of individuals in the United States. This dataset includes features such as BMI, blood pressure, age, gender, and physical activity levels, which are relevant for modeling health risks and generating exercise recommendations. When applied to the NHANES dataset, our model maintained competitive performance, with only slight variations in MAE and F1-Score. These results suggest that the model can generalize across diverse demographic groups and health conditions. The full results of the external validation on NHANES are summarized in Table 12. MIMIC-III is a large, publicly available database of ICU patients, including heart rate, blood pressure, oxygen saturation, and other clinical measurements. We applied our model to predict health risks and recommend exercise modalities for ICU patients, with adjustments made to account for the critical nature of their health conditions. The model performed well, although the performance metrics (e.g., MAE and F1-Score) were slightly lower than those achieved on the original dataset due to the more complex and varied nature of ICU patient data. This external validation suggests that while our model is robust across different populations, domain-specific adjustments may be needed for certain use cases. In both cases, the model demonstrated its potential for equitable health promotion across diverse populations, but we emphasize the need for further validation on additional datasets and real-world scenarios to fully assess its broad applicability. The claim of a “new benchmark” has been rephrased to reflect that while our model shows promising results, it requires further validation before such a claim can be substantiated.

**Table 12:** External validation results on NHANES and MIMIC-III datasets.

**Dataset**	**Model**	**MAE (mean ±SD)**	**F1-score (%)**
NHANES	Our model	13.45 ± 0.36	94.60 ± 0.25
	Random Forest	15.30 ± 0.48	92.80 ± 0.30
MIMIC-III	Our model	15.20 ± 0.42	93.80 ± 0.28
	XGBoost	16.10 ± 0.45	92.50 ± 0.32

## 4 Discussion

This section discusses the broader humanistic implications of the proposed model, interprets the key findings, and outlines the study's limitations along with avenues for future exploration.

### 4.1 Humanistic implications and strategy design for health promotion

**Figure 18:**
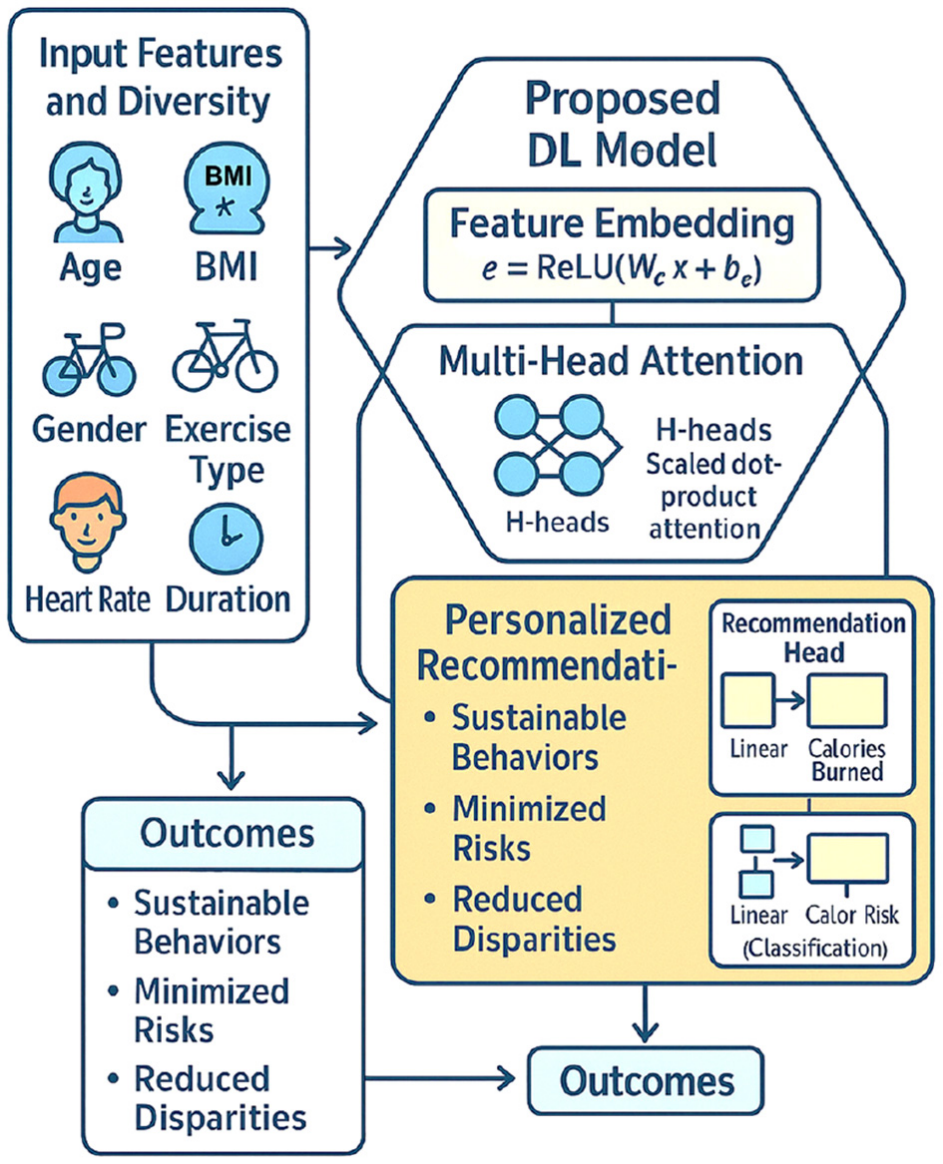
Overview of the humanistic personalized health promotion framework using deep learning.

Figure 18 illustrates the comprehensive humanistic framework underlying the proposed model. The Input Features and Diversity panel highlights the model's recognition of demographic and physiological variability. It captures key factors including Age, BMI, Gender, Exercise Type, Heart Rate, and Duration. The central Hexagonal Proposed DL Model visually decomposes into two functional modules: Feature Embedding and Multi-Head Attention mechanisms. This architecture emphasizes both mathematical transformation of features and adaptive prioritization based on their relevance to health outcomes.

Downstream, the Personalized Recommendations block delineates model outputs for both regression and classification heads. It guides individualized exercise strategies based on predicted caloric outcomes and optimal activity types. The Humanistic Fairness and Adaptation banner symbolizes the framework's overarching commitment to equitable, empathetic, and inclusive health promotion.

The proposed deep learning framework fundamentally redefines digital health intervention design by embedding personalization and fairness at its core. Rather than delivering uniform exercise prescriptions, it dynamically adjusts health recommendations according to physiological, demographic, and behavioral diversity. This adaptation supports safe, effective, and sustainable engagement in physical activities for individuals across varied fitness profiles.

Attention mechanisms within the architecture ensure that health-critical features like Heart Rate and Duration receive enhanced focus during prediction. This dynamic weighting enables nuanced adaptation to individual capabilities and exercise tolerances. Strategically, the model fosters a preventive, participatory health ecosystem. It empowers individuals to engage proactively in their own well-being and supports the broader public health goal of achieving equitable health promotion in increasingly diverse societies.

These quantitative outcomes are not merely technical improvements, they enable real-world impact in the context of human-centered health. For example, the model's high attention weights for features such as heart rate and duration indicate that these parameters are critical for generating personalized recommendations. This allows the system to adjust exercise intensity and length in a way that respects individual differences in cardiovascular endurance, fatigue thresholds, and recovery cycles. In practical terms, this means that an older adult with a high BMI will not receive the same recommendation as a younger, athletic user–even if their calorie goals are similar. By prioritizing the most physiologically relevant features through attention mechanisms, the system ensures that interventions are not only effective but also safe and inclusive. This approach minimizes the risk of over-exertion, enhances user trust, and fosters sustainable engagement. These benefits are essential in promoting equitable access to digital health services, particularly for underserved populations often excluded from one-size-fits-all models. Thus, the quantitative results directly reinforce the framework's broader goal of health for all.

### 4.2 Discussion

The experimental results validate the proposed model's capacity to outperform traditional machine learning models and shallow deep learning baselines across both regression and classification tasks. The reduction in Mean Absolute Error (MAE) and the consistent superiority in classification metrics, including Accuracy, Precision, Recall, and F1-Score, affirm the effectiveness of combining feature embedding, residual connections, and attention-driven dynamic weighting in health outcome prediction.

The use of multi-head attention was instrumental in capturing feature-specific priorities, especially emphasizing the importance of Duration and Heart Rate, which align with physiological determinants of calorie expenditure. Moreover, the stratified train-validation-test split ensured fairness across demographic groups, avoiding biases that often plague digital health tools. The incorporation of explainability through attention weights and statistical validation further enhances the transparency, accountability, and ethical acceptability of the proposed framework for real-world deployment.

The observed gains in predictive performance over state-of-the-art baselines substantiate that deep, adaptable architectures are better suited to address the complexity of human-centered exercise behavior modeling than conventional fixed-rule models. Importantly, the model's design supports both interpretability and predictive strength, bridging a critical gap between black-box AI models and actionable, understandable health guidance systems.

### 4.3 Strengthening public health contribution

To ensure that our model contributes effectively to public health, we must consider several key factors related to its deployment, user safety, behavioral adherence, and risk management in real-world applications. The deployment of the model into health interventions involves integrating it with existing digital health platforms, such as mobile health apps, fitness trackers, and telemedicine systems, which can leverage the model's ability to generate personalized exercise recommendations based on users' physiological data. We recommend a phased deployment, beginning with small-scale pilot studies to validate the model's effectiveness and gather user feedback. Real-time monitoring systems should be in place to adjust recommendations dynamically, based on ongoing user data and behavior, ensuring that the model is responsive to individual needs over time. User safety is a primary concern when offering health-related recommendations. While the model's suggestions are intended to be general guidelines, it is essential to adopt a human-in-the-loop approach for high-risk individuals, where health professionals can review recommendations before they are presented to the user. This ensures that the model's outputs align with best health practices and reduce the risk of recommending inappropriate exercise intensities, especially for users with underlying health conditions. Additionally, the model should include safeguards, such as warning systems that flag potentially harmful recommendations. For instance, when suggesting exercise routines for users with cardiovascular conditions or other health risks, the model should provide clear warnings or flag certain activities as requiring professional consultation.

In terms of behavioral adherence, encouraging users to consistently follow personalized exercise recommendations requires combining the model's outputs with behavioral strategies that promote motivation and long-term engagement. Incorporating features such as gamification, where users earn rewards for maintaining exercise consistency, can help maintain motivation. Setting realistic, achievable goals based on individual health status, and regularly adjusting these goals as users progress, will also ensure that the recommendations are both safe and sustainable. Providing personalized feedback and support through social features or direct messaging can further enhance user compliance and increase long-term engagement. Managing the risks associated with false positives and false negatives is essential for maintaining the safety and effectiveness of the model. False positives—such as recommending high-intensity exercise for individuals who may not be physically capable–can have serious health consequences, while false negatives—failing to recommend exercise for individuals who could benefit from it–may hinder potential health improvements. To mitigate these risks, the model could incorporate a confidence threshold that only provides recommendations when the model's prediction confidence exceeds a certain level. Moreover, implementing a risk assessment system where each recommendation comes with a confidence score and a risk level can help users make informed decisions. For instance, when the model suggests a specific exercise, it could indicate how confident it is in the recommendation, and provide a risk level (e.g., low, medium, or high) based on the user's health profile and the confidence of the prediction. These considerations will help ensure that the model is not only effective but also safe and reliable for use in real-world health interventions. By addressing deployment pathways, user safety, behavioral adherence, and risk management, we aim to enhance the model's potential for equitable health promotion, ensuring that its recommendations are both actionable and appropriate for a diverse range of users.

### 4.4 Limitations and future directions

While the proposed model demonstrates strong predictive performance and humanistic fairness, several limitations warrant attention. First, the dataset, although rich in exercise features, does not include broader behavioral, nutritional, or psychological variables that also influence exercise effectiveness and health trajectories. Incorporating multimodal health data, such as dietary logs, stress levels, and wearable sensor outputs, could further improve personalization. Second, the current framework treats health promotion as a static prediction problem, whereas real-world health behavior is dynamic and context-dependent. Future extensions should explore reinforcement learning approaches or longitudinal deep learning models that can adapt recommendations over time based on evolving participant data and feedback. Third, while fairness across gender and age was considered, more sophisticated fairness audits across intersectional identities (e.g., ethnicity, socioeconomic status) are necessary before deploying the model widely. Additionally, external validation on more diverse, global datasets would help ascertain generalizability across different populations. Lastly, although the attention mechanism improves model interpretability, future work could integrate explainable AI (XAI) techniques such as SHAP values or counterfactual explanations to offer even deeper insights into model behavior for end-users and health professionals. Thus, future research should focus on enhancing contextual adaptability, integrating broader health determinants, and embedding explainability more deeply into digital health architectures.

Another important limitation concerns the representativeness of the dataset. While the Exercise and Fitness Metrics Dataset provides rich information across a range of demographics, its user base is primarily drawn from publicly available activity tracking applications, most of which are popular in Western countries. As a result, the data may underrepresent individuals from non-Western regions, particularly those with differing cultural attitudes toward exercise, varying access to digital fitness tools, or alternative health norms. This geographic bias limits the generalizability of the model's recommendations to global populations. It also poses challenges in ensuring fairness across socio-cultural contexts, where physiological responses or exercise patterns may differ significantly. Future work should incorporate more globally representative datasets, including those from underrepresented regions, to ensure the model's humanistic goals are met at scale and across diverse health environments.

## 5 Conclusion

This study proposed a fairness-aware, attention-driven deep learning framework for personalized digital health interventions. Moving beyond predictive accuracy, the model integrates humanistic principles by emphasizing adaptability, equity, and interpretability.

In terms of practical deployment, the proposed model can be embedded in mobile fitness applications or wearable device platforms to deliver personalized exercise prescriptions based on real-time physiological inputs. Its interpretable structure–particularly the attention-based prioritization of features like heart rate and duration–allows for user-centered feedback, which is critical in building trust and encouraging sustained engagement in physical activity. From a policy perspective, this framework could inform population-level preventive health programs by enabling scalable, data-driven personalization without sacrificing fairness. Health institutions and public health agencies may leverage this model to develop inclusive digital health platforms that adapt to diverse demographic profiles, particularly in communities with limited access to in-person healthcare resources.

Future development should focus on expanding the model to integrate behavioral and environmental data streams, as well as validating its performance across culturally diverse populations. These efforts will further enhance its role as a practical and ethical tool in the evolving landscape of digital health.

## Data Availability

The original contributions presented in the study are included in the article/supplementary material, further inquiries can be directed to the corresponding author.
